# Supplementary figures and images for: Neural manifold under plasticity in a goal driven learning behaviour
Source: PLoS Comput Biol. 2021 Feb 5;17(2):e1008621. doi: 10.1371/journal.pcbi.1008621 (PMC7864452; doi:10.1371/journal.pcbi.1008621)

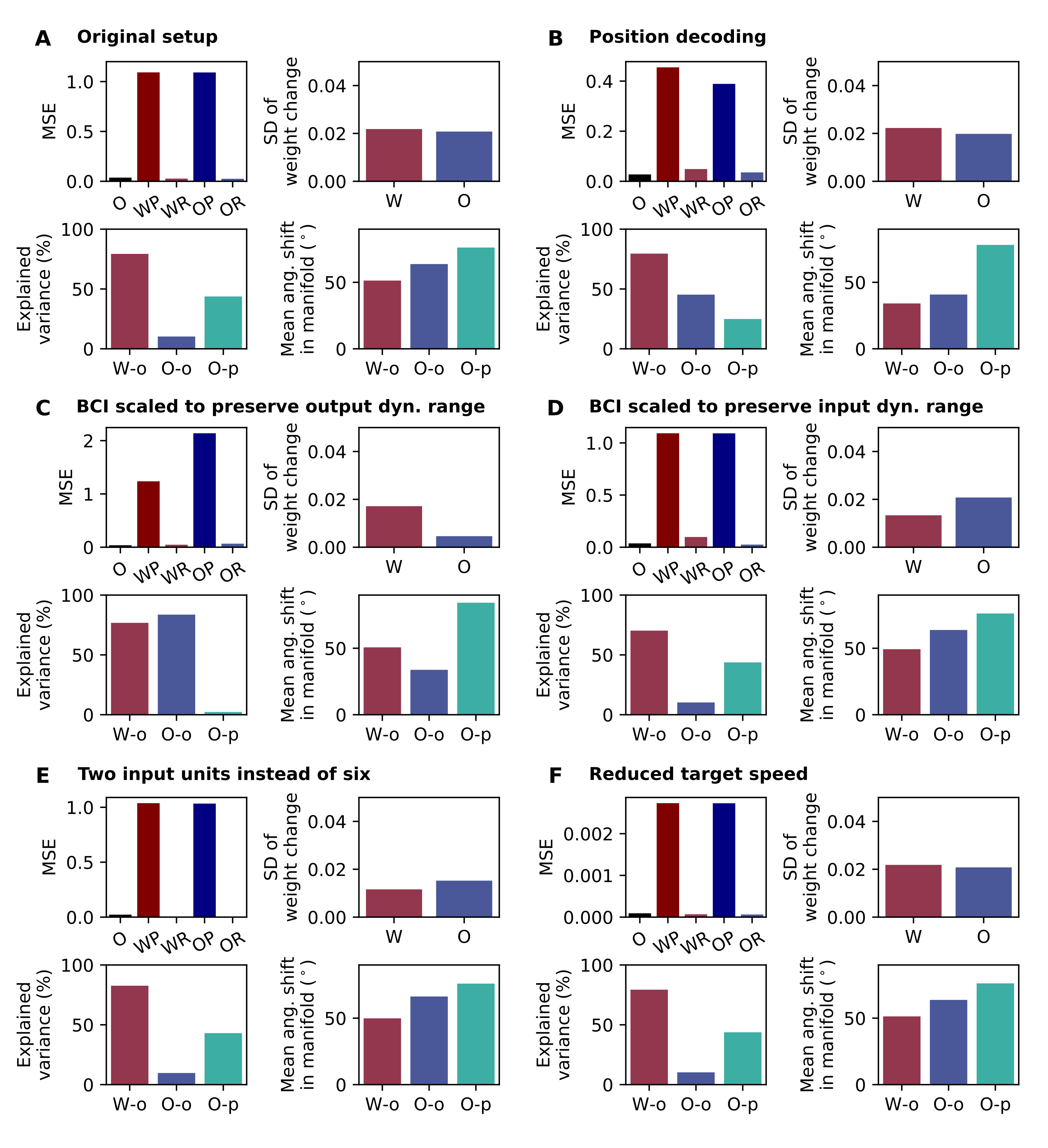

Supplement: S1 Fig — (A) Original setup as in Fig 2. Upper left panel shows task performance, measured as mean squared error (MSE), after initial training (O), after within-manifold perturbation (WP), after within-manifold retraining (WR), after outside-manifold perturbation (OP) and after outside-manifold retraining (OR). Upper right panel shows the standard deviation of the weight change distribution between before and after retraining for within- (W) and outside-manifold (O) retraining. Lower left panel shows the manifold overlap between initial and retrained manifold for within- (W-o) and outside-manifold (O-o) perturbation, as well as the overlap between retrained and target manifold for outside-manifold perturbations (O-p). Lower right panel shows the mean principal angle between the same manifold as in lower left panel. (B) Position decoding instead of velocity decoding. (C) BCI perturbations are scaled in order to preserve the range of velocities after perturbation. This especially affects outside-manifold perturbations as in this case the velocity values are normally only 10% of the original ones, due to a signal loss caused by the outside-manifold projection. (D) BCI perturbations are scaled to assure that the theoretical approximation for the neural activity after retraining, given the initial training state, does not exceed the dynamic range of the neurons. (E) Instead of having one input unit for each target, here, there are only two input units. They signal x and y position of the targets. (F) Reduced target speed of 0.01, instead of 0.2. (PNG) [file pcbi.1008621.s001.png]

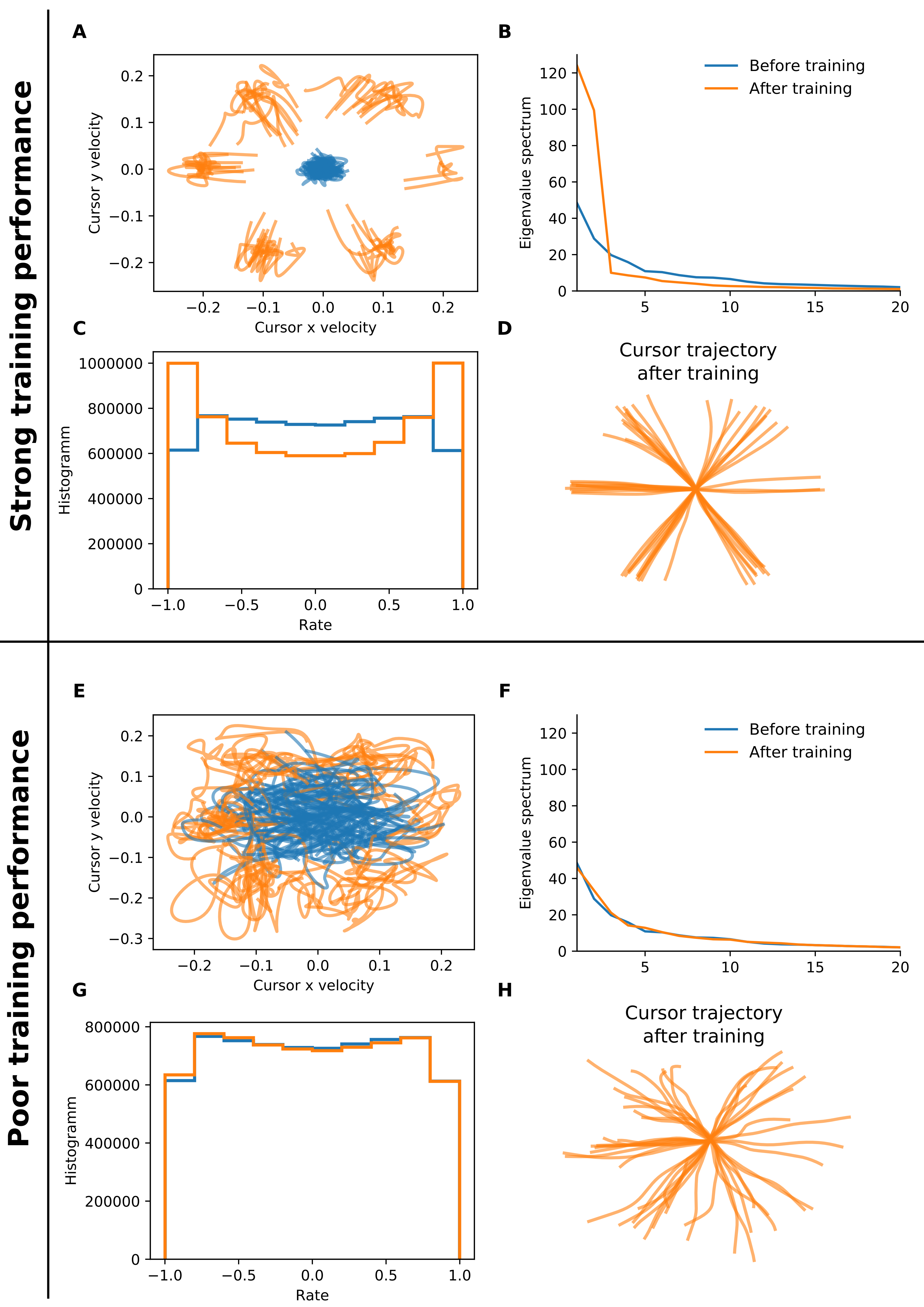

Supplement: S2 Fig — (A-D) Initial training with random decoder, normalized and scaled with factor 0.04 (the default version used in the paper). (E-H) Initial training with random decoder, normalized and scaled by a factor 0.2. (A) and (E) Cursor velocities before and after training. (B) and (F) Eigenvalue spectrum of network dynamics before and after training. (C) and (G) Rate distribution before and after training. (D) and (H) Reconstructed cursor trajectory after training. (PNG) [file pcbi.1008621.s002.png]

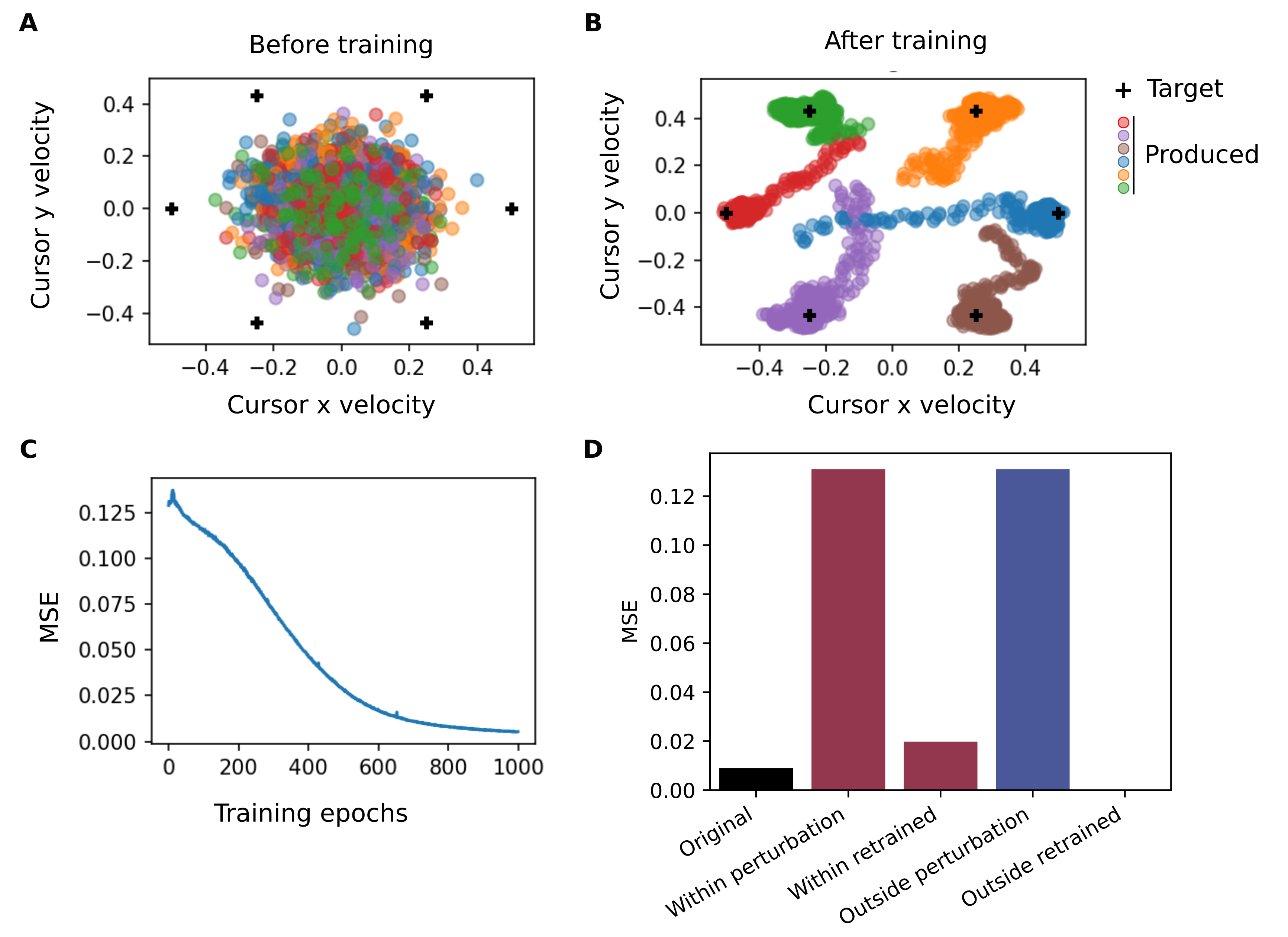

Supplement: S3 Fig — Here, the network dynamics are given by ht = tanh(Wih xt + Whh h(h−1)). Each trial has 100 time steps and the target cue is given during the first 5 steps. The input xt is modelled similar to our main setup. The stimulus amplitude is 50. The incoming weights Wih are fixed and randomly drawn from a uniform distribution between −1/N and 1/N, where N is the number of neurons in the network. The recurrent weight matrix is initialized in a similar fashion as in our main setup, except that here, we use a fully connected network. For gradient descent we use Adam optimizer [52] with learning rate 0.001. The loss and the fixed output decoder is the same as in our main setup. The training uses an ideal-observer feedback signal to propagate the error in cursor velocities to errors on single neurons in the recurrent network. For training, we use a batch size of 30. (A) Cursor velocities before initial training. (B) Cursor velocities after initial training. (C) Learning curve for initial training. (D) Performance results for within- and outside-manifold retraining. Number of training epochs for initial training, as well as retraining, is 1000. The MSE value in (C-D) is not summed over time or trials. (PNG) [file pcbi.1008621.s003.png]

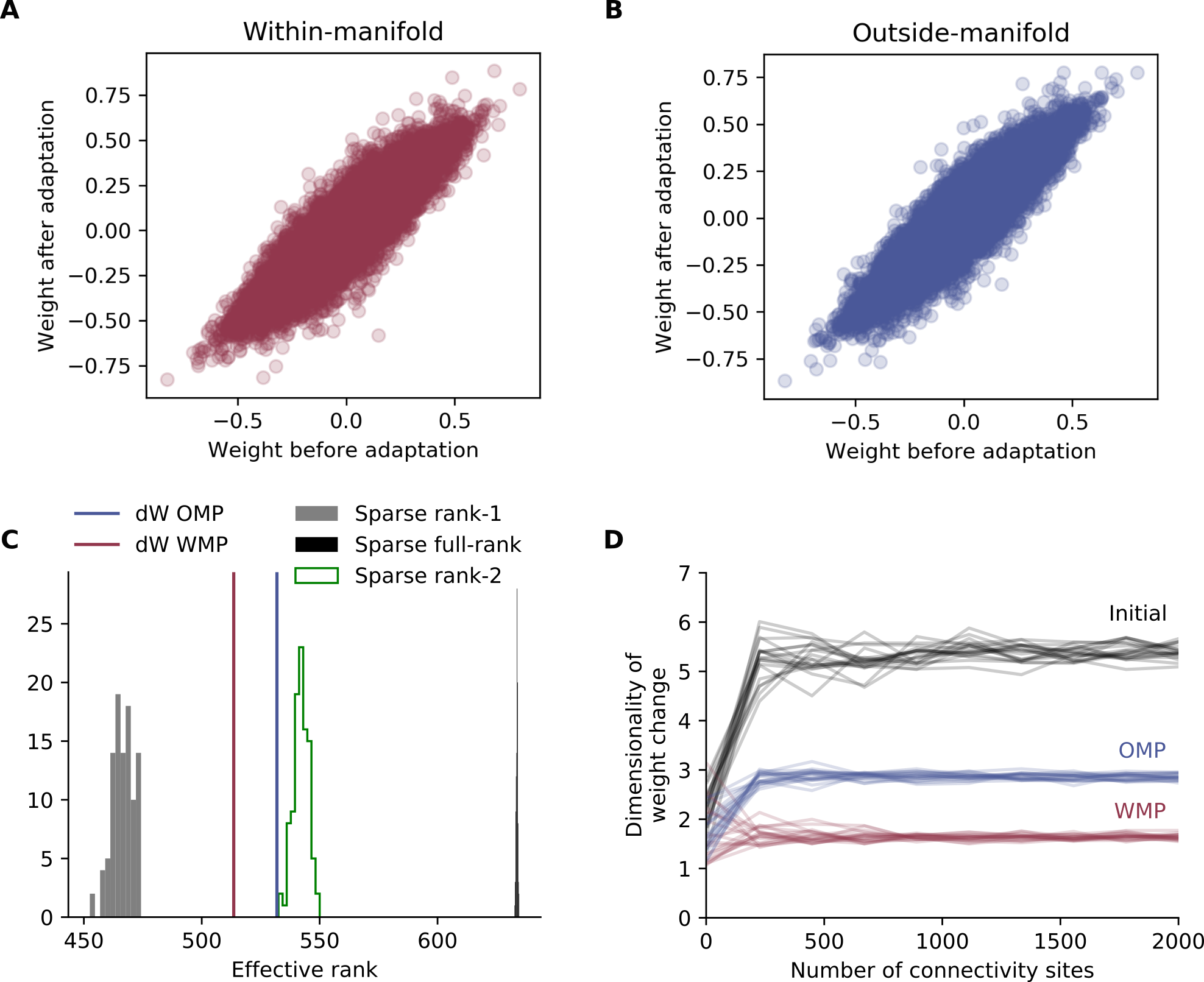

Supplement: S4 Fig — (A) Relation between weight before retraining and after retraining for within-manifold perturbation. (B) Relation between weight before retraining and after retraining for outside-manifold perturbation. (C) Measurement of the effective rank [53] of the weight change matrix for within-manifold and outside-manifold retraining. Both values are compared to values obtained for random rank-1, random rank-2 and full-rank matrices, having the same sparsity as the network weight matrix. (D) Dimensionality of weight change dynamics during retraining (dW measured at the end of each trial). Since it is not feasible to calculate the full covariance matrix for all plastic connections, as the number is too high (∼ 64000), we calculated the dimensionality from randomly chosen subsets of connections and checked for convergence. (PNG) [file pcbi.1008621.s004.png]

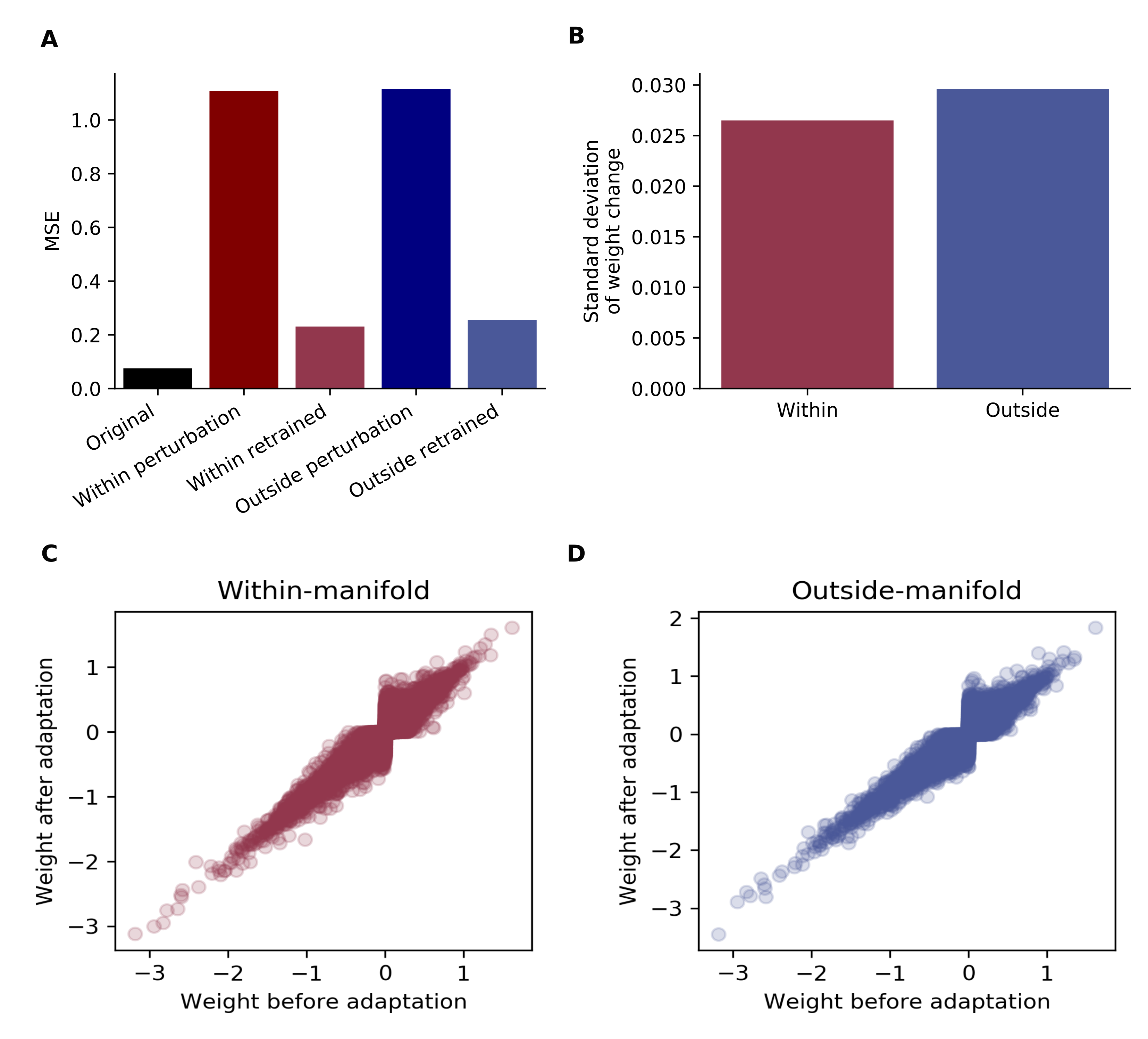

Supplement: S5 Fig — Simulation results for a network with Daleian connectivity. Each neuron has either positive (excitatory) or negative (inhibitory) outgoing weights. During learning, the sign of these weights is preserved. If a learning step would produce a sign flip we instead clip the weight to zero. (A) Training performance measured as mean squared error (MSE). (B) Standard deviation of weight change during within- and outside-manifold training. (C) and (D) Weights before versus after within- (C) or outside- (D) manifold training. (PNG) [file pcbi.1008621.s005.png]

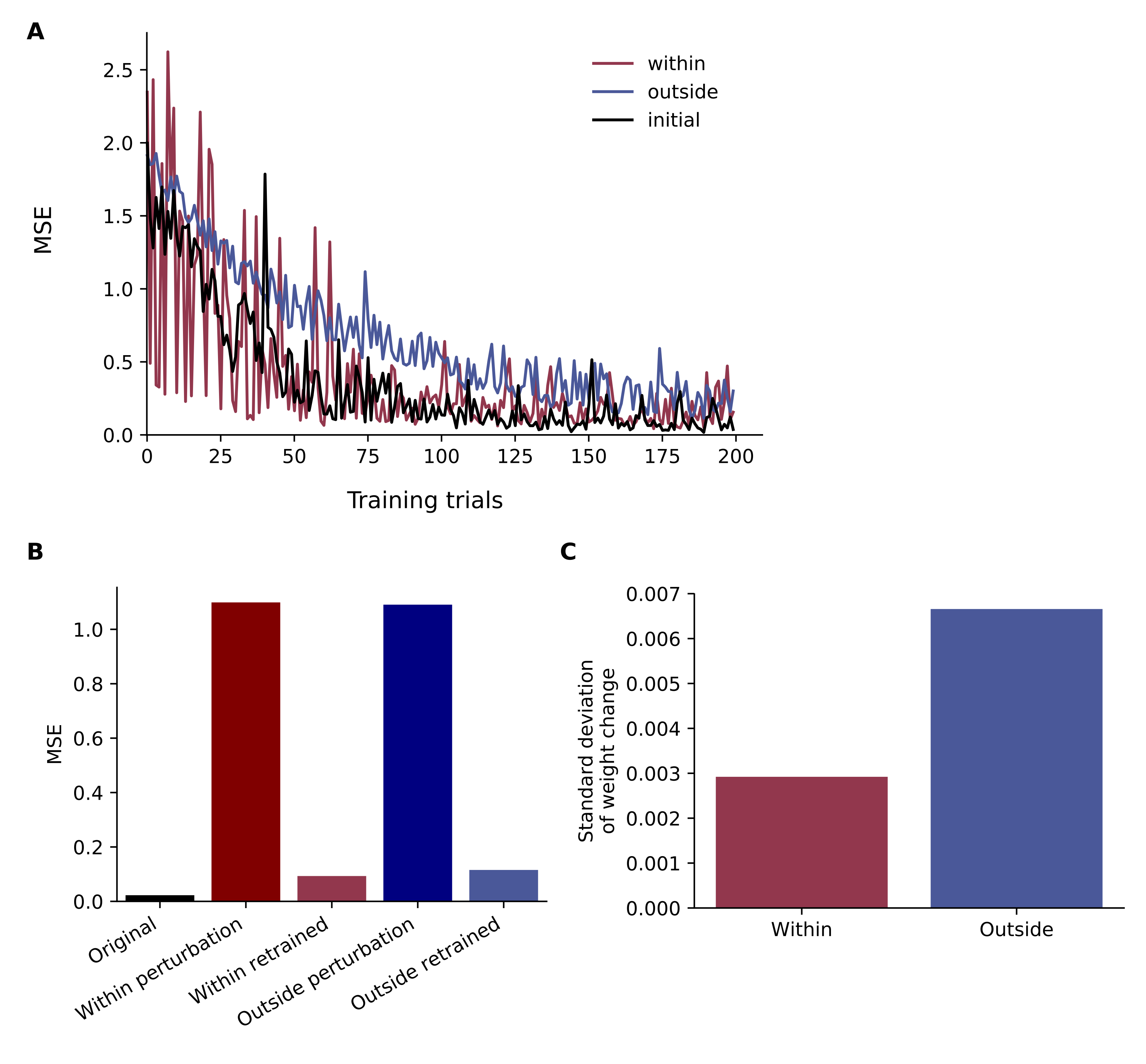

Supplement: S6 Fig — Implementation of recently proposed local learning rule approximating backpropagation-through-time algorithm [47]. The weight update is given by dWijt=-ejt(1-tanh2(xjt))∑t′≤t-1rit′, where ej is the error for neuron j, xj is the activity of neuron j and ri = tanh(xi) is the rate of neuron i. (A) Learning curves for initial, within- and outside-manifold training period. Here, the mean squared error (MSE) is summed over update steps per trial, which are 90 steps. (B) Performance results after training measured as MSE. (C) Standard deviation of weight change during within- and outside-manifold training. (PNG) [file pcbi.1008621.s006.png]

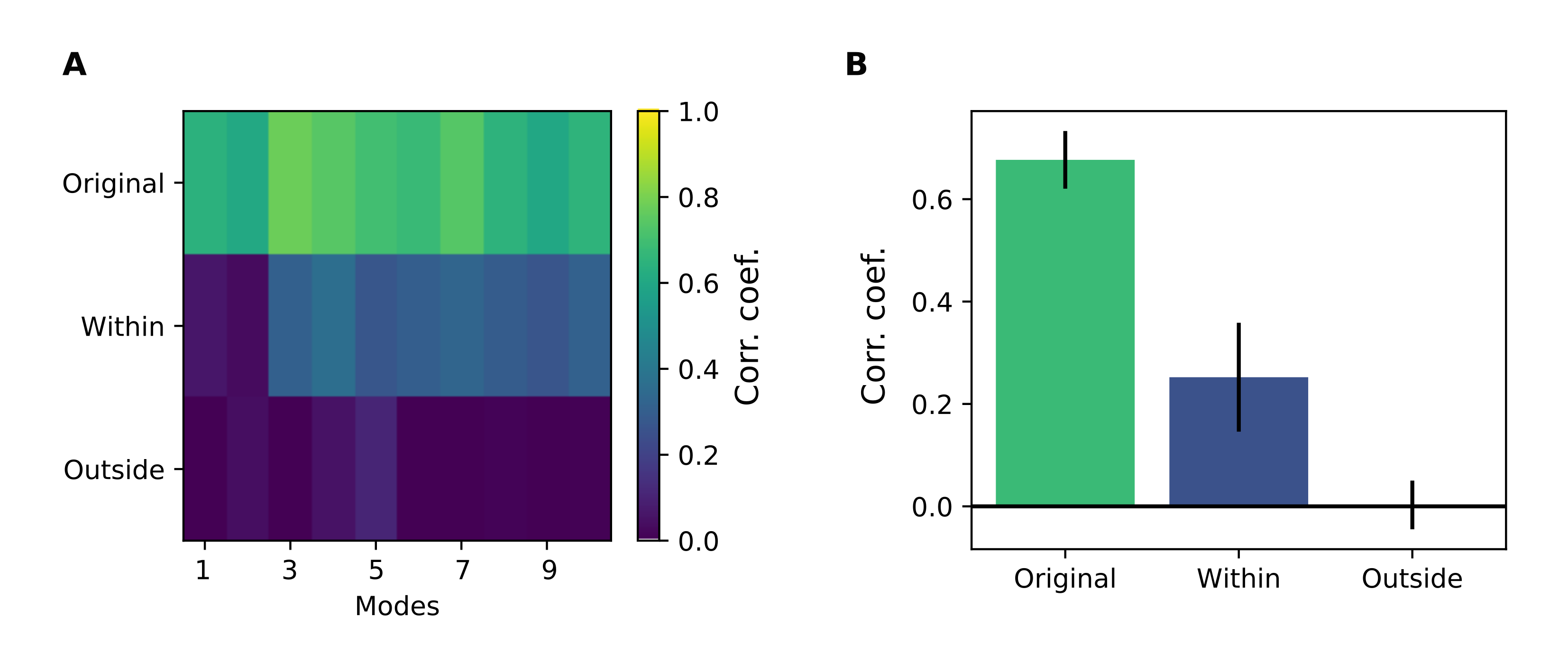

Supplement: S7 Fig — (A) Correlation between the neural modes before and after retraining for each of the six targets. Note, the neural manifold is in this case not the actual internal manifold (which is different after retraining), but the static one defined by the initial BCI mapping. We averaged over 20 simulations and the 6 targets. (B) Average mode correlation. Error bars are standard deviation across the ten modes. (PNG) [file pcbi.1008621.s007.png]

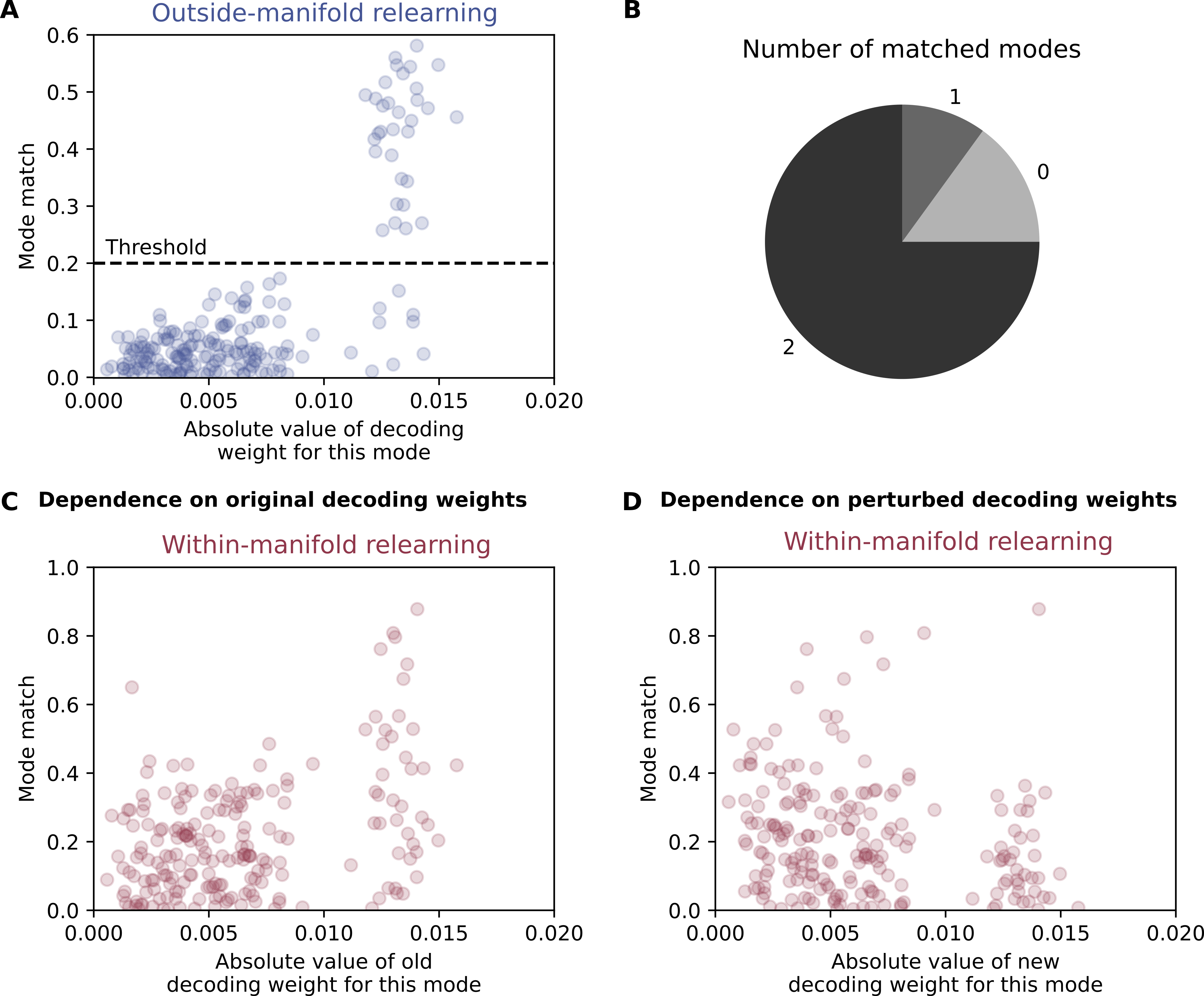

Supplement: S8 Fig — (A) Mode match is defined as scalar product between the Eigenvector of the specific Eigenvalue before and after retraining. Each dot represents the mode match of one of ten Eigenvalues considered, and shows the result for one of 20 simulation runs (total 200 points shown). We defined a mode as matched if the scalar product is bigger than 0.2. (B) We quantified how many modes are matched per simulation run, taking the definition from (A). (C) Same as in (A), but now for within-manifold retraining. As the decoding weights are shuffled during a within-manifold perturbation one can compare the mode match to the decoding weights before and after perturbation. Here, we compared to before perturbation. (D) Mode match for within-manifold retraining compared to decoding weights after perturbation. (PNG) [file pcbi.1008621.s008.png]

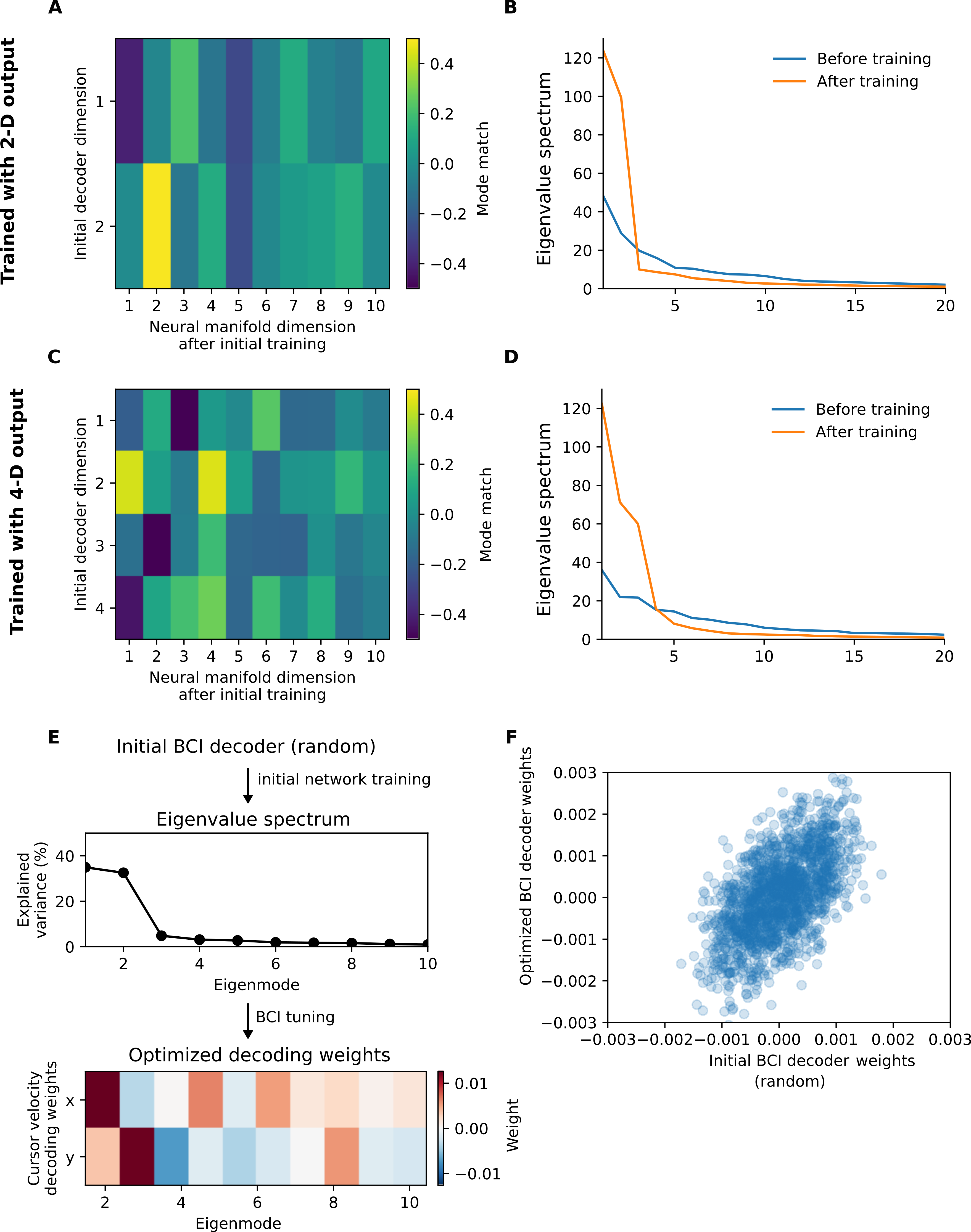

Supplement: S9 Fig — (A-B) Standard case where initial random decoder output is two-dimensional. (A) Mode match is calculated as scalar product between vectors. The initial decoder induces the first two strong neural modes, which are strongly amplified in the Eigenvalue spectrum after initial training (B). (C-D) Test case where initial random decoder output is four-dimensional. Here, the assignment of initial decoder and neural manifold modes is less prominent, meaning that there is no one mode specifically responsible for one output dimension. (C). In this case the number of amplified modes does not correspond to the dimensionality of the initial decoder (D) (E) Initial random decoder shapes Eigenvalue spectrum during initial network training phase, and Eigenvalue spectrum in turn shapes optimized BCI decoding weights D. (F) Relation between optimized BCI decoder and initial random one used for initial network training. (PNG) [file pcbi.1008621.s009.png]

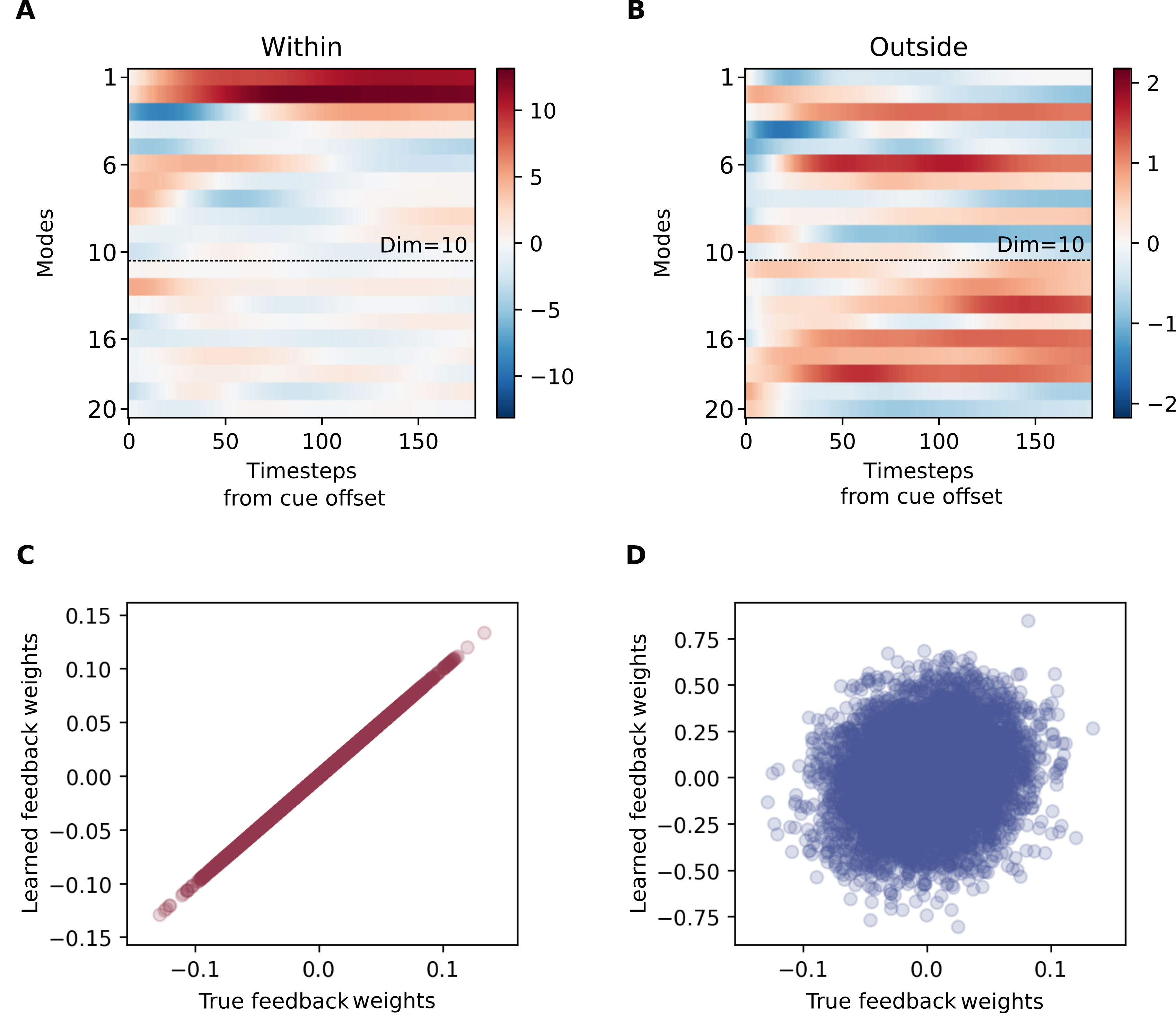

Supplement: S10 Fig — (A-B) Mode activation during one example trial for a within-manifold perturbation (A) and an outside-manifold perturbation (B). Mode activation is obtained by projecting measured neural dynamics onto the original neural manifold, given by the transformation matrix C. (C-D) Results of linear regression to infer inverse of transformation matrix C for a within-manifold perturbation (C) and an outside-manifold perturbation (D). (PNG) [file pcbi.1008621.s010.png]

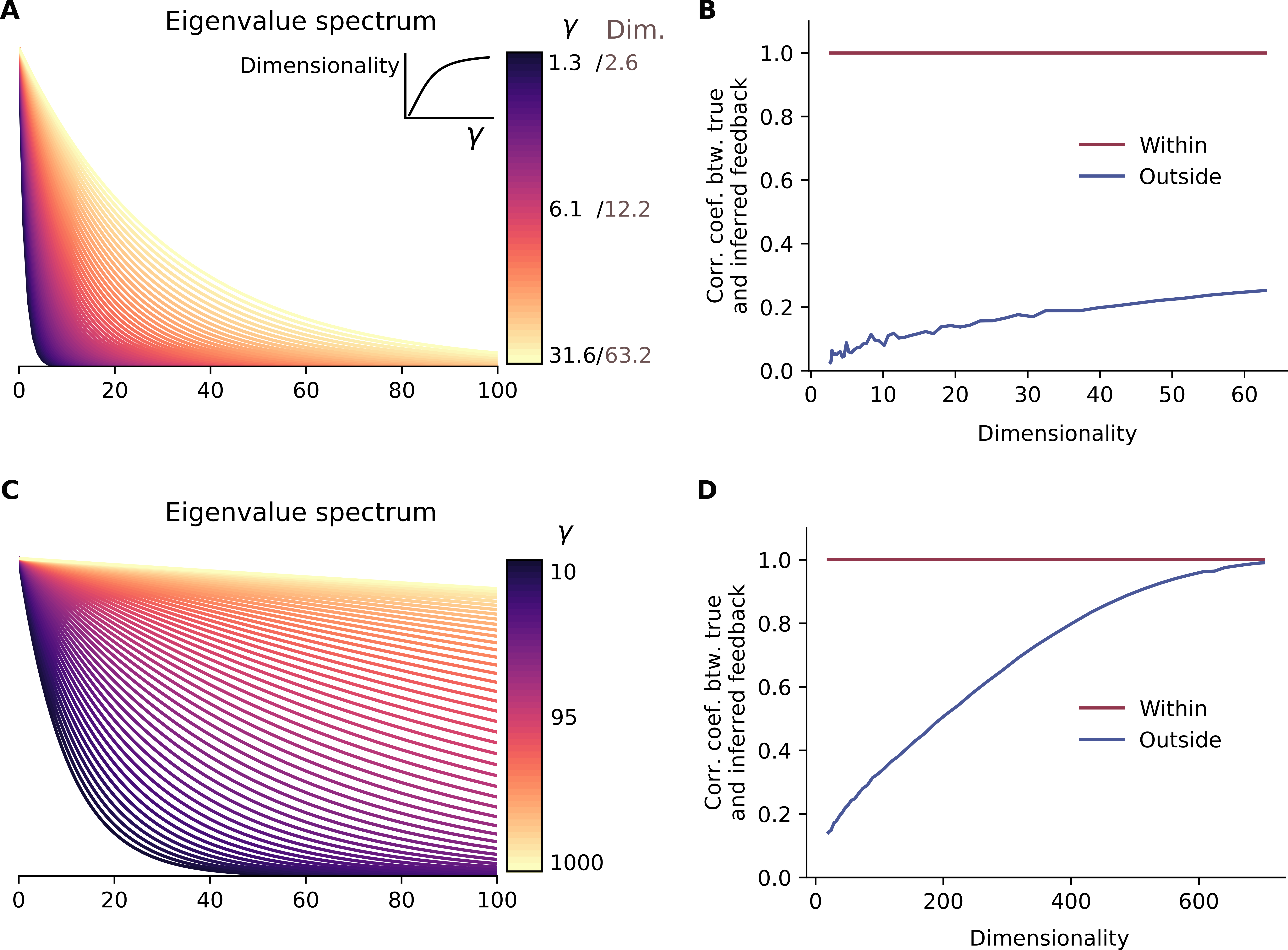

Supplement: S11 Fig — (A) and (C) Imposed Eigenvalue spectra, defined by f(x;γ) = exp−x/γ. Dimensionality is calculated by (∑iλi)2/∑i(λi2) where λi is the ith Eigenvalue. Detailed methods are described in S1 Appendix (B) and (D) Feedback learning results measured by correlation coefficient between inferred and true feedback weights, dependent on the dimensionality of the imposed Eigenvalue spectrum. (PNG) [file pcbi.1008621.s011.png]

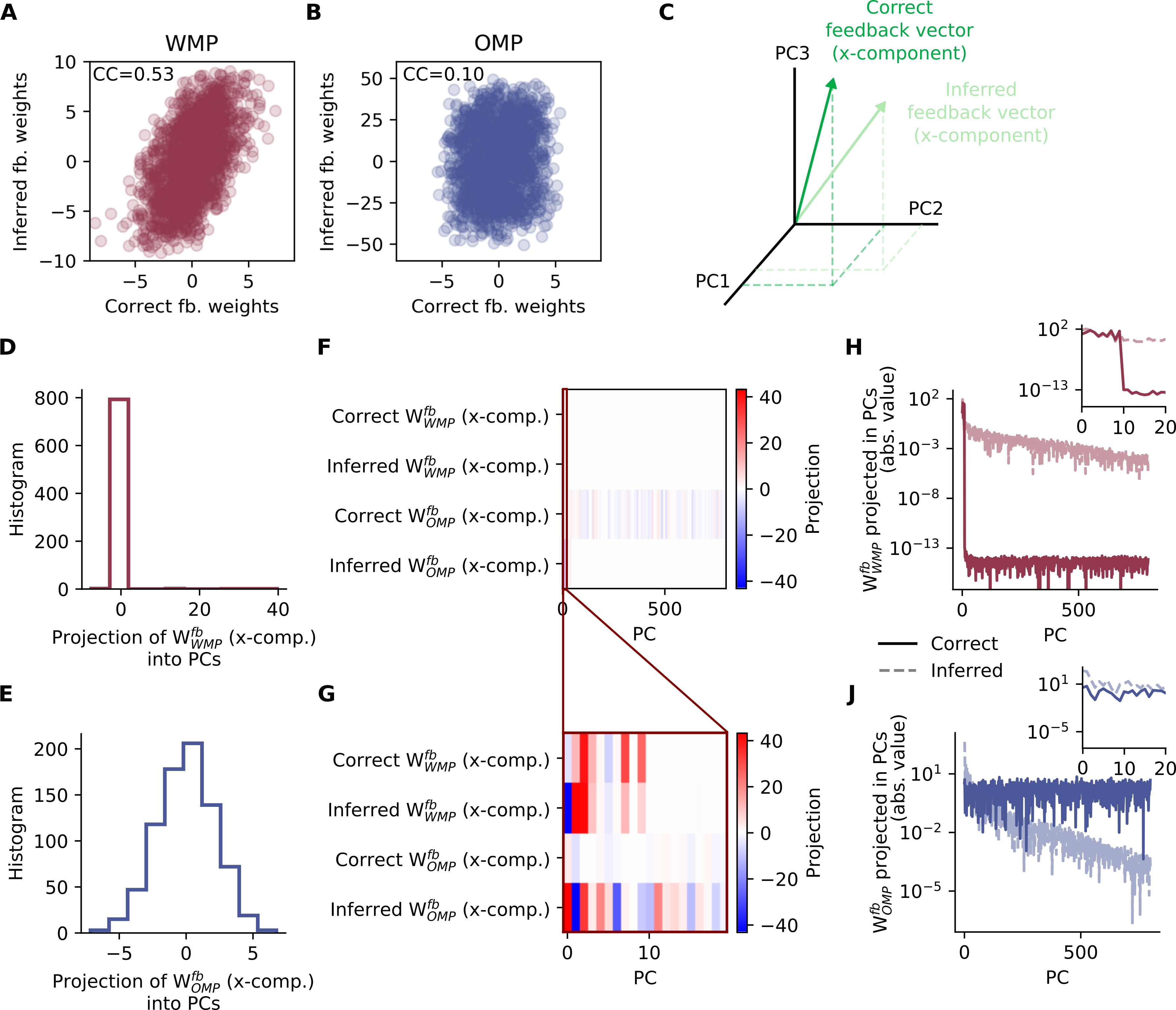

Supplement: S12 Fig — (A-B) Feedback learning results for within-(WMP) (A) and outside-manifold perturbation (OMP) (B). CC describes the correlation coefficient between inferred and correct feedback weights. Same data as in Fig 4B and 4C. (C) Feedback weight vectors for x- and y-component of cursor dynamics can be expressed in terms of principal components of neural dynamics. With this, we can compare feedback learning performance, dependent on the related neural mode. Per definition, the most prominent neural modes in the system lie in the first principal component axes. (D-E) Distribution of projections into the principal component basis for correct feedback (x-component) for within-manifold perturbation (D) and outside-manifold perturbation (E). (F) Comparison of projections into the principal component basis for correct and learned feedback vectors (x-component). (G) Same as (F) but zoomed in to show only the first 20 principal components. (H-J) Absolute value of projection into principal component basis for inferred and correct feedback weights for within- (H) and outside-manifold perturbation (J). The inlets show the same plot as (H-J), but focussing on principal components 1-20. (PNG) [file pcbi.1008621.s012.png]

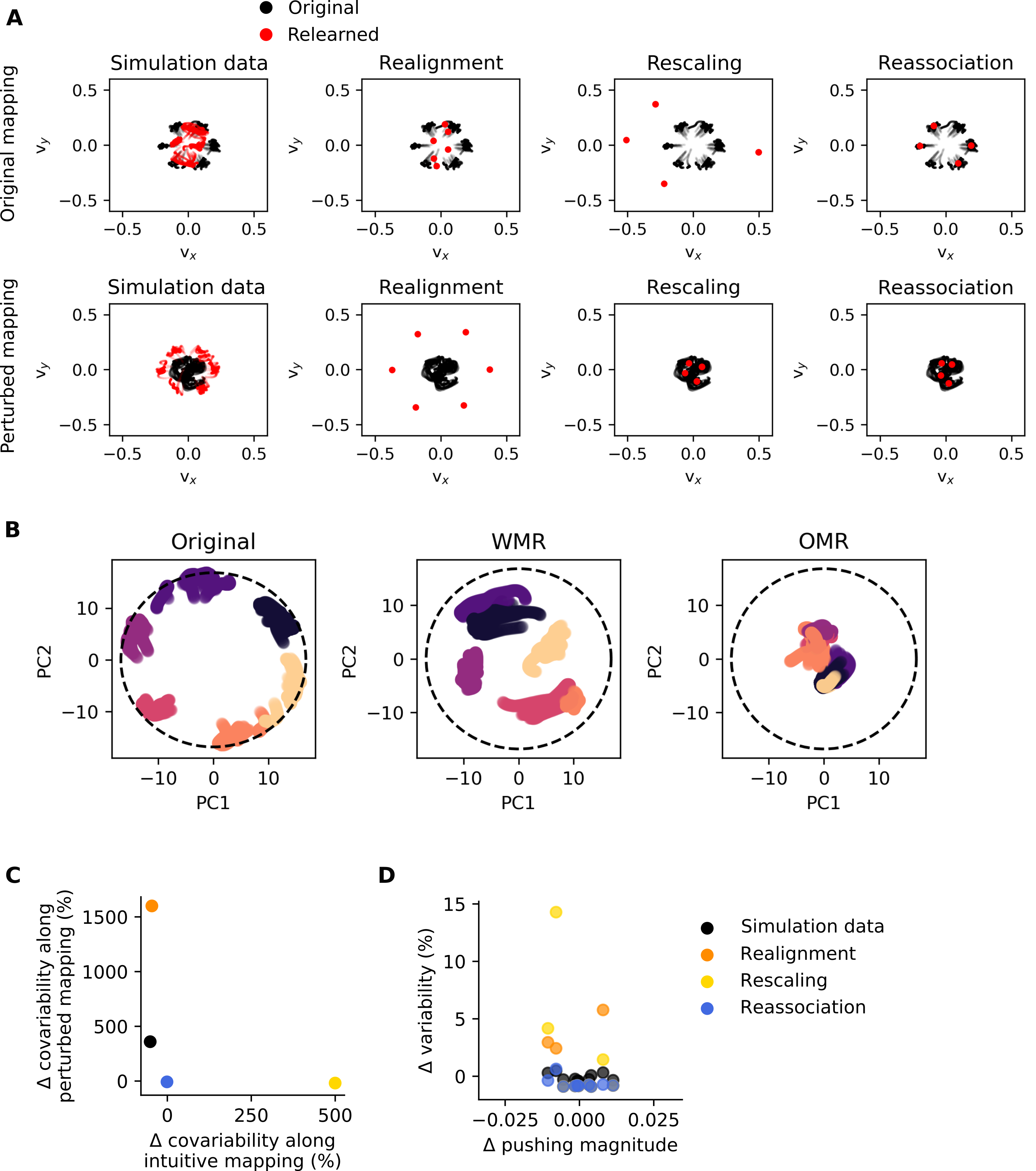

Supplement: S13 Fig — (A) Cursor velocities under original (upper row) and perturbed mapping (lower row) for original (black) and retrained network (red). Comparison between simulation data (first column), realignment hypothesis (second column), rescaling hypothesis (third column) and reassociation hypothesis (fourth column) [49]. Detailed methods are described in S2 Appendix. B) Original neural activity (left), neural activity after within-manifold relearning (middle) and neural activity after outside-manifold relearning (right) (cf. Fig 2). Neural activity is projected into the original neural manifold and the first two principal components are shown. Different colors correspond to different target locations. Dashed black circle represents boundary of initial repertoire. (C) Change in covariability along original and perturbed mapping. (D) Change in variability of neural modes dependent on the imposed change in the BCI readout (pushing magnitude). (PNG) [file pcbi.1008621.s013.png]

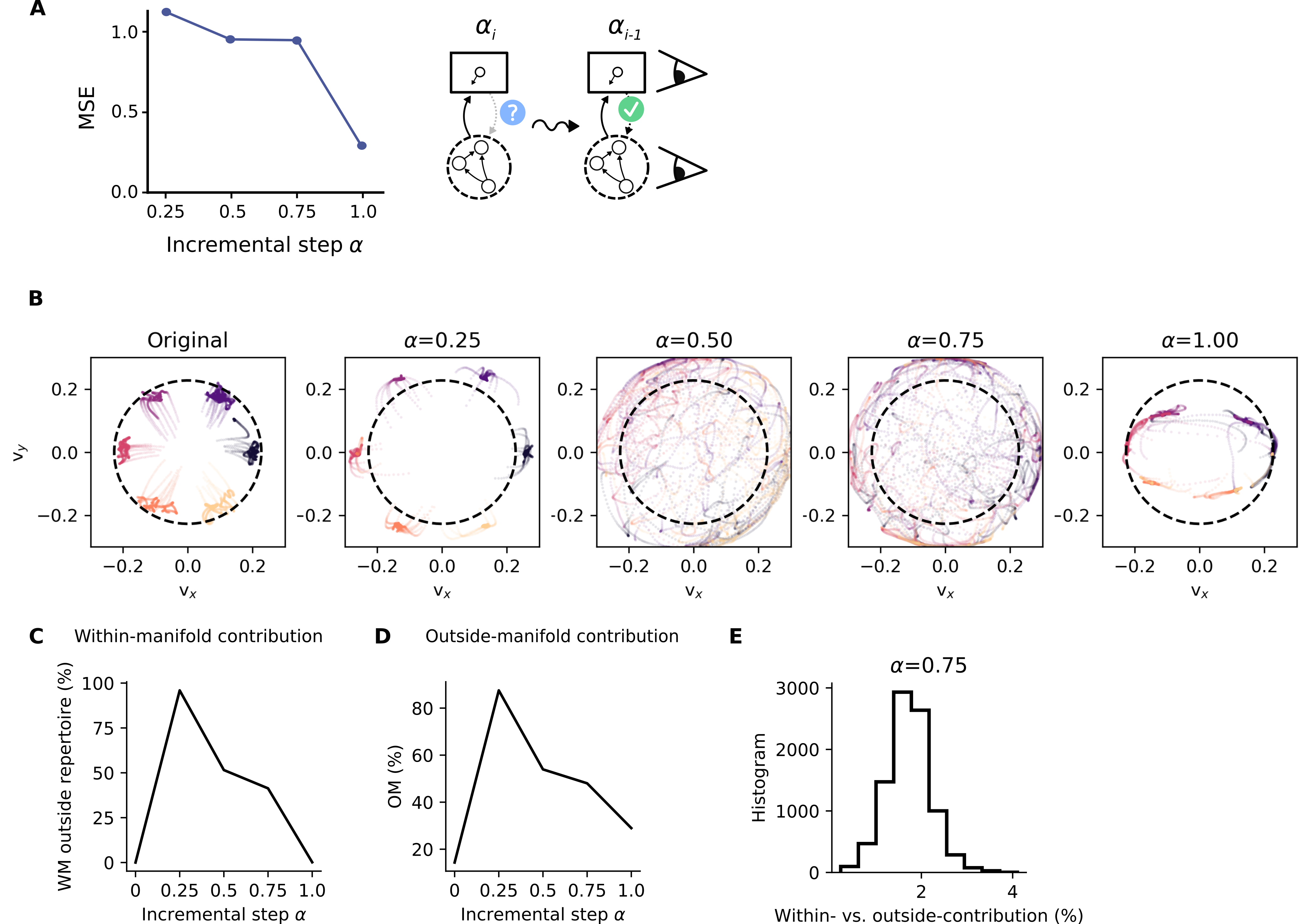

Supplement: S14 Fig — (A) Relearning performance under incremental strategy (Fig 5E). (B) Cursor velocities under the original mapping for each incremental training step. Dashed black circle shows boundary of initial repertoire. Colours represent different targets. (C) Percentage of within-manifold patterns which lie outside of the original repertoire (dashed black circle in (B)). (D) Percentage of variability measured outside of original manifold as a function of incremental step α. (E) Histogram of percentage of within- versus outside- manifold contributions. For each relearned neural activity pattern which was outside of the original manifold or repertoire we calculated the projection onto the original manifold dWM (within-manifold contribution) and subtracted this projection from the total neural activity vector to obtain the residual vector dOM (outside-manifold contribution). This gave two distances in the high dimensional space of neural activity. To calculate the percentage of within-manifold contribution we calculated dWM2/(dWM2+dOM2). (PNG) [file pcbi.1008621.s014.png]

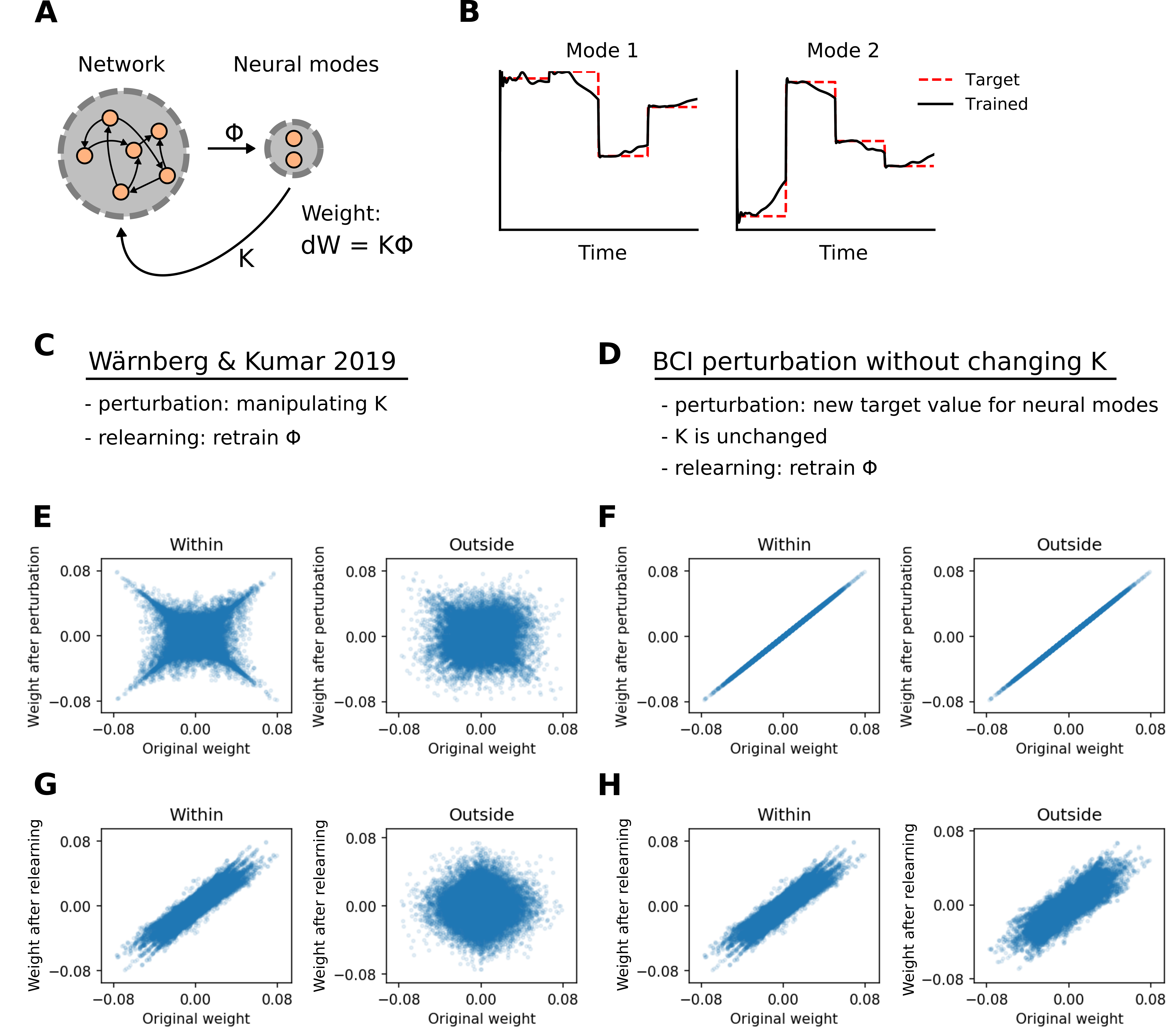

Supplement: S15 Fig — (A) Reimplementation of Waernberg and Kumar [34]. Neural modes are decoded from a random recurrent network with static weights. The neural mode signal is fed back into the recurrent network via a feedback matrix K. Network training consists of finding the decoding weights ϕ which produce a given target signal at the neural mode level. FORCE is used to adapt the decoding weights accordingly [17]. (B) Resulting neural mode dynamics after initial training. (C) To implement the BCI experiment from Sadtler et al. Waernberg and Kumar manipulated the feedback matrix K and retrained the decoding weights ϕ in order to produce the original target dynamics on the mode level [34]. (D) Alternative implementation of the BCI perturbation which is closer to our simulations. Here, the BCI perturbation is implemented by setting a new target signal on the mode level, without manipulating K. (E) Weight change after perturbation, without retraining, for implementation of Waernberg and Kumar. (F) Weight change after perturbation, without retraining, for alternative implementation. (G) Weight change after retraining for implementation of Waernberg and Kumar. (H) Weight change after retraining for alternative implementation. (PNG) [file pcbi.1008621.s015.png]

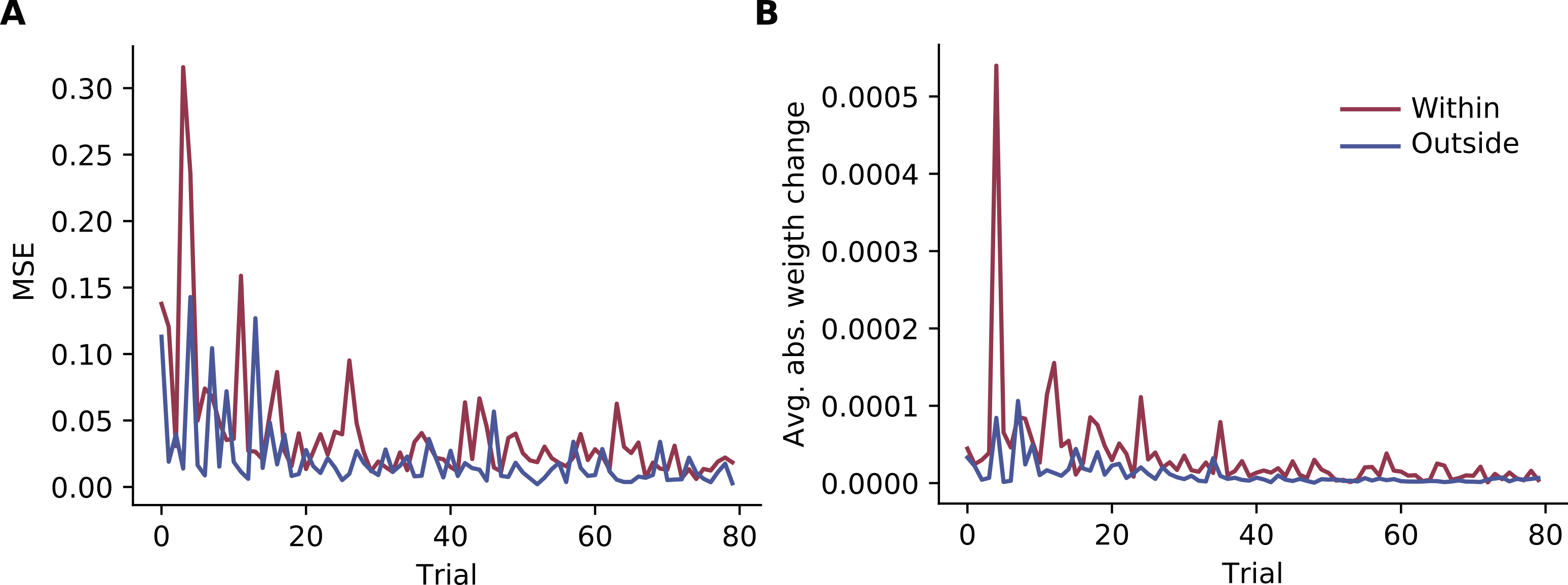

Supplement: S16 Fig — (A) Mean squared error (MSE) during relearning for within- and outside-manifold perturbation. The MSE is summed over all update steps in a trial, which constitutes 90 steps. (B) Amount of weight change measured during relearning for within- and outside-manifold perturbation. (PNG) [file pcbi.1008621.s016.png]

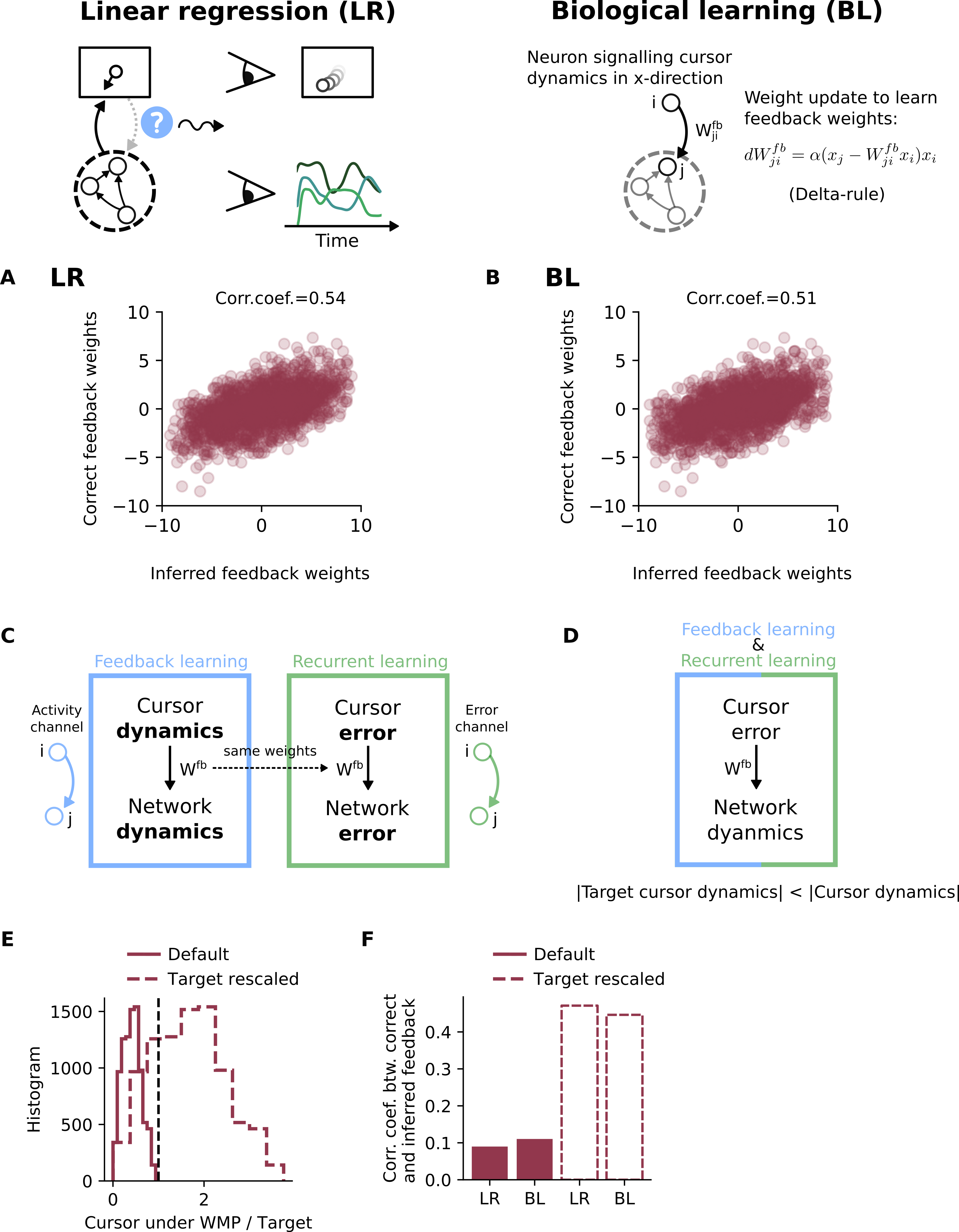

Supplement: S17 Fig — (A) Inferred feedback weights using linear regression (LR) are compared to correct feedback weights, which are given by the pseudo-inverse of the BCI mapping. In linear regression, weight factors are estimated to predict neural dynamics from cursor dynamics. (B) Linear regression can be implemented by a simple, biologically plausible, learning rule (BL). Learned feedback weights, using a biological learning rule, are compared to correct feedback weights. (C) Illustration of relearning steps: 1) Feedback weights are inferred from observing neural and cursor dynamics. 2) The learned feedback weights (Wfb) are then used to propagate the error form cursor to network level. (D) Alternative way of inferring feedback weights. Instead of predicting neural dynamics from cursor dynamics, neural dynamics could also be predicted by the observed error signal in cursor dynamics. (E) Cursor dynamics under within-manifold perturbation are in general smaller than the target values. Rescaling target values by 0.25 (dashed line) reverses the relation. (F) Feedback learning performance, measured as correlation coefficient between correct and inferred feedback weights, when cursor error signal is used to predict neural dynamics. (PNG) [file pcbi.1008621.s017.png]

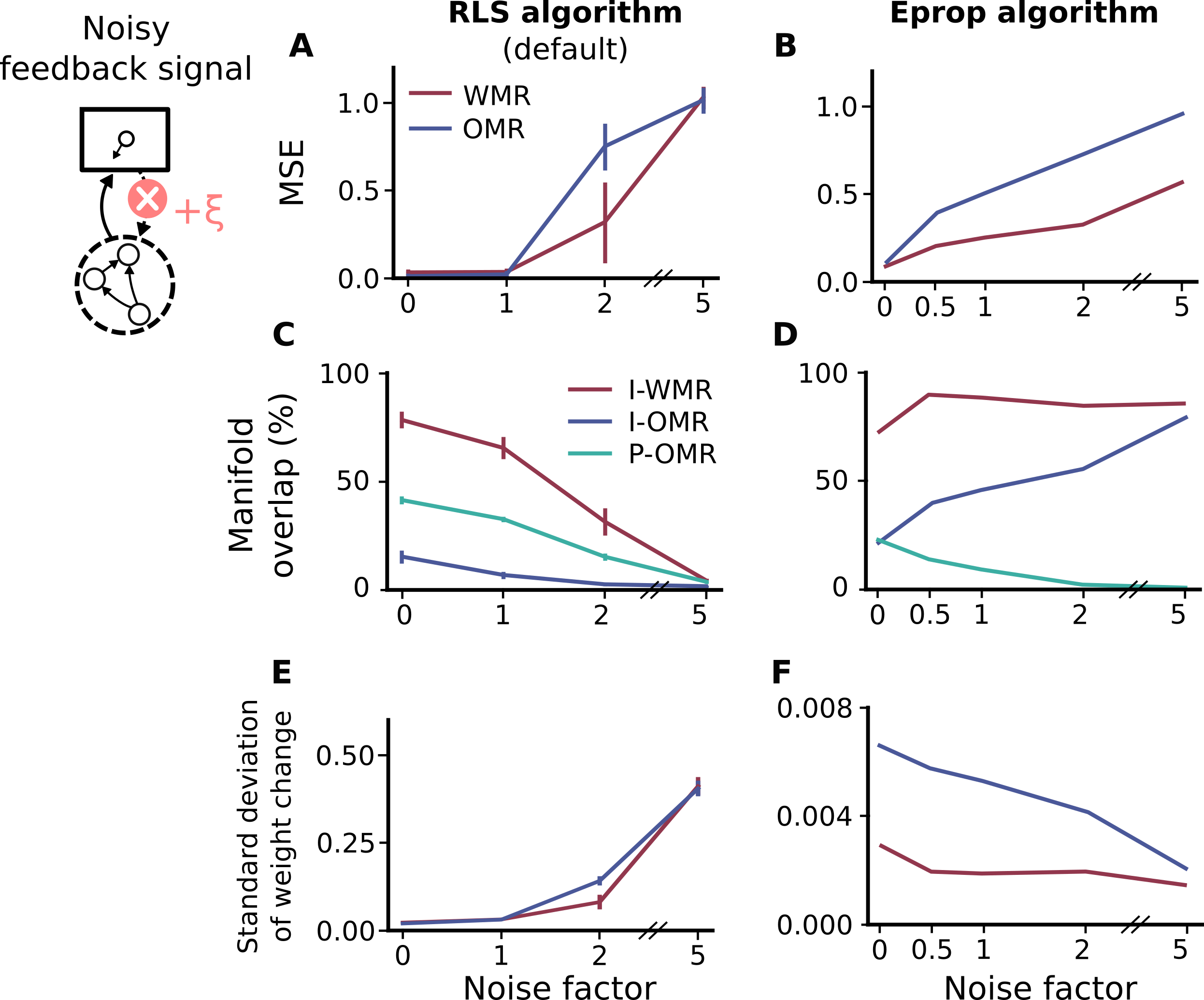

Supplement: S18 Fig — (A-B) Relearning performance, measured as mean squared error (MSE), as a function of the amplitude of the noise in the feedback signal using recursive-least-squares (RLS) algorithm (A) and an alternative implementation with a local learning algorithm (Eprop) (B). (C-D) Manifold overlap between original manifold and manifold after within learning (I-WMR), original manifold and manifold after outside learning (I-OMR) and manifold defined by BCI perturbation and manifold after outside learning (P-OMR). (E-F) Weight change during relearning. (A,C,E) Simulations with RLS (same as Fig 3B in the main paper). (B,D,F) Simulations with Eprop learning rule. (PNG) [file pcbi.1008621.s018.png]

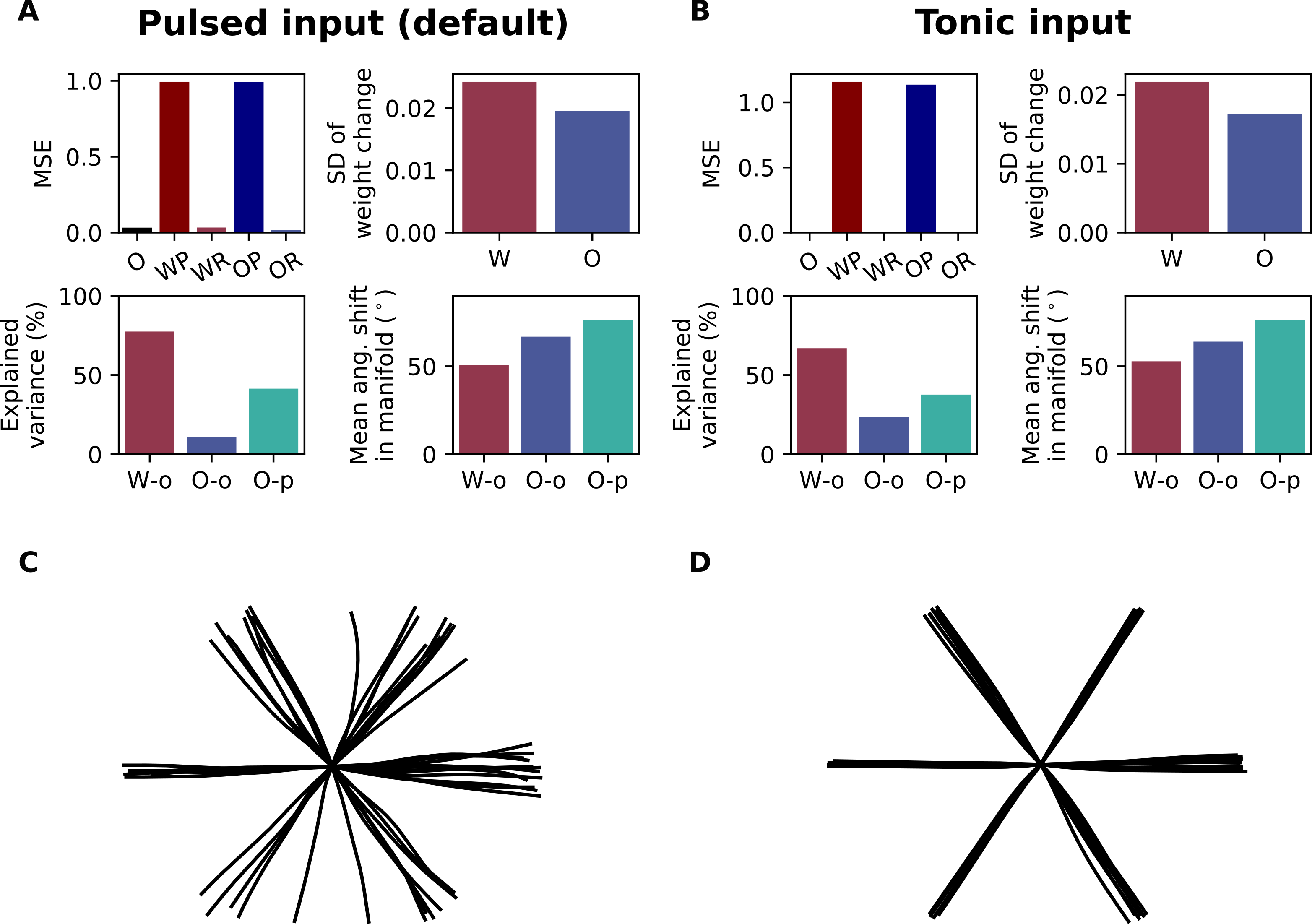

Supplement: S19 Fig — (A-B) Upper left panel shows task performance, measured as mean squared error (MSE), after original training (O), after within-manifold perturbation (WP), after within-manifold retraining (WR), after outside-manifold perturbation (OP) and after outside-manifold retraining (OR). Upper right panel shows the standard deviation of the weight change distribution between before and after retraining for within- (W) and outside-manifold (O) retraining. Lower left panel shows the manifold overlap between original and retrained manifold for within- (W-o) and outside-manifold (O-o) perturbation, as well as the overlap between retrained and target manifold for outside-manifold perturbations (O-p). Lower right panel shows the mean principal angle between the same manifold as in lower left panel. (A) Result for simulations where inputs are modelled as a pulse (same as S1A Fig). (B) Results for simulations where inputs are modelled as tonic input. (C-D) Reconstructed cursor trajectories for pulsed input simulations (C) and tonic input simulations (D). (PNG) [file pcbi.1008621.s019.png]

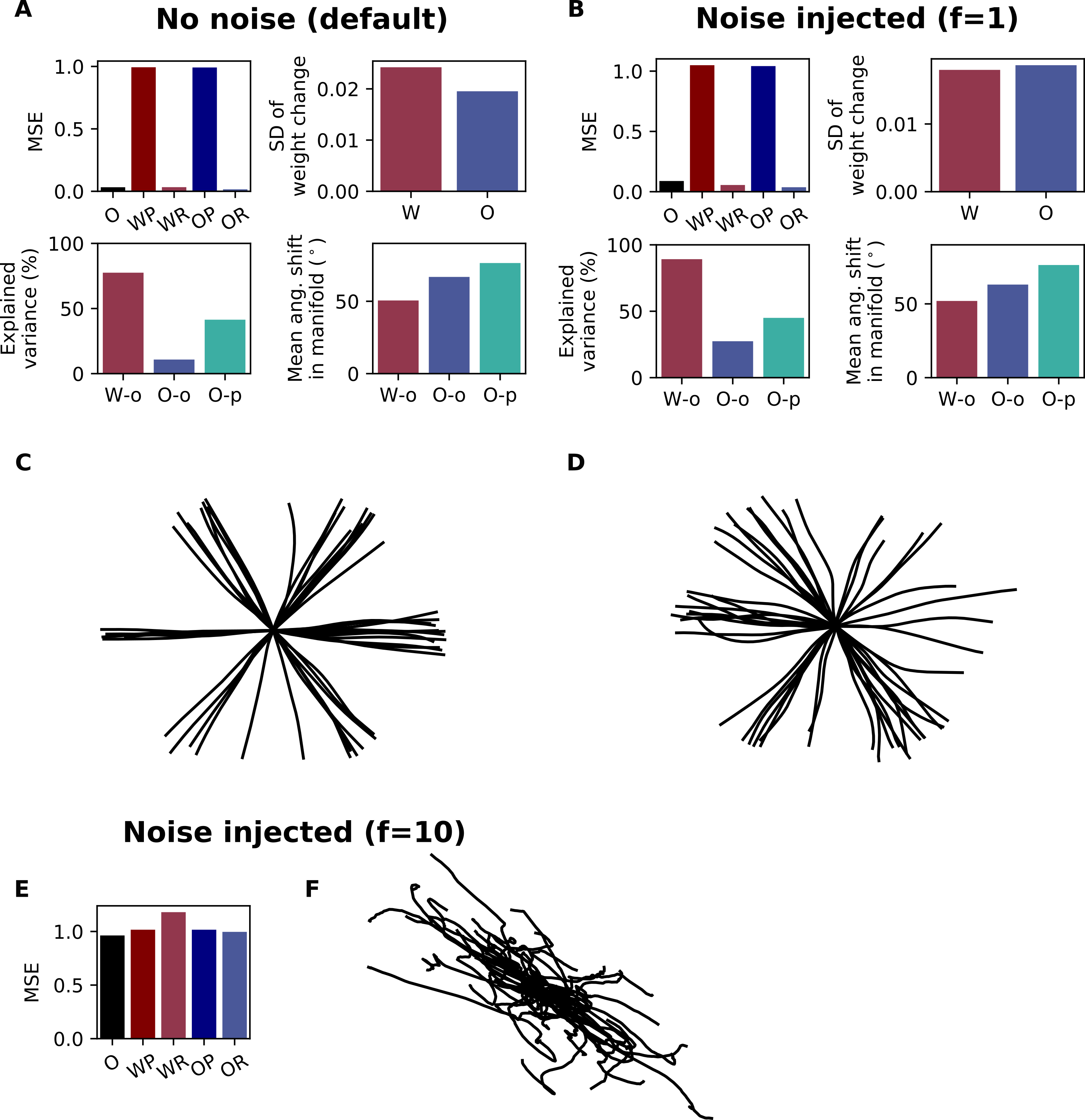

Supplement: S20 Fig — (A-B) Upper left panel shows task performance, measured as mean squared error (MSE), after original training (O), after within-manifold perturbation (WP), after within-manifold retraining (WR), after outside-manifold perturbation (OP) and after outside-manifold retraining (OR). Upper right panel shows the standard deviation of the weight change distribution between before and after retraining for within- (W) and outside-manifold (O) retraining. Lower left panel shows the manifold overlap between original and retrained manifold for within- (W-o) and outside-manifold (O-o) perturbation, as well as the overlap between retrained and target manifold for outside-manifold perturbations (O-p). Lower right panel shows the mean principal angle between the same manifold as in lower left panel. (A) Standard simulations where no noise is injected (same as S1A Fig). (B) At each time step each neuron receives an extra input, drawn from a normal distribution with zero mean and standard deviation f. The noise for each neuron is independent. (C) Reconstructed trajectories for simulations without noise. (D) Reconstructed trajectories when noise (f = 1) is injected. (E-F) Network cannot be trained to perform the task if noise is too high (f = 10). (PNG) [file pcbi.1008621.s020.png]
