# Supplementary material for: Neural manifold under plasticity in a goal driven learning behaviour
Source: PLoS Comput Biol. 2021 Feb 5;17(2):e1008621. doi: 10.1371/journal.pcbi.1008621 (PMC7864452; doi:10.1371/journal.pcbi.1008621)
Supplement: S1 Appendix — (PDF) [file pcbi.1008621.s021.pdf]

Simplified model to investigate feedback learning

In order to understand why it is possible to learn feedback weights for a within-manifold perturbation, but not for an outside-manifold one, we developed and analysed a simplified setup. We created a synthetic dataset, which has no temporal structure, but is simply drawn from a multivariate Gaussian distribution. The number of variables was matched to the number of neurons in the simulated network, such that there was 800 random variables ξ in total. Without loss of generality, we set the mean to zero, as we wanted to analyse the effect of the covariance structure on the ability to perform feedback learning. To create the covariance matrix $\mathbf{\Sigma}$ of the synthetic data, we firstly created the desired Eigenvalue spectrum which is given by

$$f(x;\gamma) = \exp^{-x/\gamma}$$

where γ parametrizes how fast the spectrum decays and $x = 0$ correspond to the first Eigenvalue, $x = 1$ to the second and so forth. After setting the diagonal values of the covariance matrix, we performed a basis transformation which puts the synthetic data in the same Eigenspace as found in the simulated data.

$$\mathbf{\Sigma} = C^T \operatorname{diag}(f(0;\gamma), f(1;\gamma), ..., f(n-1;\gamma))C$$

$$\xi \sim \mathcal{N}((0, 0, ..., 0), \mathbf{\Sigma})$$

To simulate a within- or an outside-manifold BCI transformation, we used the Eigenvector space C of the data and either took the first d Eigenvectors (within-manifold) or the first d Eigenvectors after shuffling the entries of each vector (outside-manifold). For Fig.S11 the number of modes d - which are decoded - is set to 10 to match the number of modes used in the main paper.
