# Supplementary material for: Neural manifold under plasticity in a goal driven learning behaviour
Source: PLoS Comput Biol. 2021 Feb 5;17(2):e1008621. doi: 10.1371/journal.pcbi.1008621 (PMC7864452; doi:10.1371/journal.pcbi.1008621)
Supplement: S2 Appendix — (PDF) [file pcbi.1008621.s022.pdf]

Adaptation of analysis performed in Golub et al. 2018

To test whether our simulation results for within-manifold learning are in line with further experimental observations (Golub et al. 2018), we performed an additional analysis dissecting the strategy the neural network is using to compensate for a within-manifold perturbation. In the original paper, Golub et al. focused on three different hypotheses: 1) Realignment, 2) Reassociation and 3) Rescaling. They constructed neural activity patterns according to these three different types of neural adaptation and compared it to their experimental recordings. We followed a similar path, constructing artificial data according to the three hypotheses and comparing it to our simulation data. The methods to create artificial data according to a given hypothesis are adapted from the original paper (finding the target-specific mean under a certain hypothesis) (Golub et al. 2018) and will briefly be explained below.

Realignment

To find the neural activity patterns which would correspond to the realignment hypothesis we searched each target for patterns that maximize the cursor velocity along the specific target direction, yet still obey the initial boundaries of neural activity in our model (-1,1). As a result, we obtained an estimate for the adapted mean neural activity pattern per target under the realignment hypothesis.

Reassociation

Under the reassociation hypothesis, adapted neural activity patterns were found by selecting from the set of initial activity patterns the best pattern for the current target and under the current perturbation. What is optimized here is again the target-specific cursor velocity, as described before. Solving this constrained optimization problem leads to estimates for the target-wise mean neural activity pattern under the reassociation hypothesis.

Rescaling

Finding the estimates for the target-wise mean neural activity pattern under the rescaling hypothesis is similar to the reassociation hypothesis. Here however, initial neural activity patterns are rescaled according to the change in BCI readout, before the constrained optimization is performed. The scale factor for neuron i is given by $f_i = \frac{\|T_{:,i}\|_2}{\|T^{WM}_{:,i}\|_2}$ where T is the BCI mapping before perturbation and T^{WM} is the BCI mapping after the within-manifold perturbation.
