# Supplementary material for: Neural manifold under plasticity in a goal driven learning behaviour
Source: PLoS Comput Biol. 2021 Feb 5;17(2):e1008621. doi: 10.1371/journal.pcbi.1008621 (PMC7864452; doi:10.1371/journal.pcbi.1008621)
Supplement: Attachment — Submitted filename: revision_letter.pdf [file pcbi.1008621.s023.pdf]

We thank the reviewers for their highly relevant and constructive feedback. We think these comments have greatly improved our manuscript, which includes new simulations and nine new supplementary figures, as well as substantial rewriting of the text. In the following, we describe how the comments and concerns raised by the reviewers are addressed.

Reviewer #1: Summary

In this manuscript, the authors use a recurrent neural network (RNN) modeling approach to investigate the origins of the different types of learning observed in a series of recent BCI studies. The key experimental result from these prior studies is that neural activity decoded from primary motor cortex of behaving monkeys can be adjusted rapidly (i.e., within a single behavioral session) following a BCI perturbation if the new desired patterns of activity respect the pre-existing covariations within the decoded neural population (so-called within-manifold perturbation, or WMP). If not (i.e., in the case of outside-manifold perturbation, OMP), learning is much slower and requires an iterative training approach that tries to minimize violations in the natural covariation structure of neural activity at every step of the procedure.

The present study is potentially appealing in that it provides one explanation for why this dichotomy might exist. Using an RNN model trained on the same task used in the origin BCI study, the authors explore the hypothesis that learning performance may depend on the nature of the synaptic weight changes required by WMP versus OMP. First, they show that both types of perturbations could in theory be learned equally well in the ideal case where weights are allowed to change directly based on feedback in order to maximize performance. Second, they assess how WMP and OMP can be learned in more biologically-plausible conditions, i.e., with imperfect feedback or sparse plasticity. Under these conditions, WMP appears to be generally more learnable than OMP. Third, they test how well the model can infer feedback signals from its own internal dynamics. Again, in this case, WMP appears to outperform OMP. Finally, they show that OMP learning can be improved if the model is trained in a stepwise manner reminiscent of what was done in the original BCI studies.

Overall impression

The manuscript is written clearly, and most claims are appropriately supported by modelling data. The authors make a reasonable case that, should learning indeed engage plasticity mechanisms within M1, the stark differences between WMP and OMP learning observed in BCI studies could in principle result from imperfect feedback information driving incomplete plastic changes. One potential concern is that in their investigation, the authors completely overlook the possibility that learning may occur upstream of the decoded neural population, and hence may not require any recurrent connectivity changes within M1, particularly for WMP. Although the authors do mention this possibility in the discussion section, the fact that it is not at all explored in the model slightly undermines the impact of the study. Moreover, it is unclear how the results favoring WMP versus OMP learning can be explained by the learning strategy used to update RNN weights during

retraining. Indeed, because the feedback learning is based on regressing the network internal dynamics against cursor movements, it seems that WMP may be trivially at an advantage. Finally, it remains unclear how the different types of feedback signals, even in their most sophisticated forms considered at the end of manuscript, could indeed operate inside the brain. Nevertheless, assuming that BCI learning is indeed related to connectivity changes, not to upstream inputs, and driven by feedback signals that can somehow be inferred from behavior, this study provides a characterization of this specific learning regime.

We appreciate the reviewer's concerns expressed here and we tried to improve our manuscript with respect to the three major concerns raised here:

1) Learning upstream: It is true that we excluded this hypothesis purposefully from our analysis in this paper. This is definitely one of the most probable option of how motor learning could be implemented in the brain, as we also mention in the discussion of our manuscript. Yet, differentiating whether learning is implemented upstream or locally in motor cortex (or both) is a highly non-trivial problem and demands – in our opinion – a whole study on its own. In contrast, we wanted to focus on one hypothesis in this paper and test to which extent it is in line with experimental observations. The fact that it is very hard to distinguish where learning is happening is highlighted by several findings in our manuscript. Firstly, we show that local plasticity does not necessarily lead to a change in neural manifold. On the contrary, due to the constraint on learning a feedback signal, the neural manifold is naturally preserved during learning (mentioned in the Discussion). Secondly, a new analysis showed that even in our implementation of learning we find that within-manifold learning is best described by reassociation (new supplementary figure S13), although reassociation has mostly been linked to a change in input drive (Golub et al. 2018).

2) Unclear explanation of relearning with a learned feedback signal: We thank the reviewer for pointing out the poorly explained procedure of recurrent learning with a learned feedback signal. To address this concern we included two new supplementary figures (Fig.S12&Fig.S17) providing further explanations of the feedback learning procedure. We also rewrote the relevant part of the manuscript to clarify our results.

3) Potential implementation in the brain: This was also a very valuable comment and we wrote a new paragraph at the end of the feedback learning section, as well as adding a supplementary figure (Fig.S12), to provide a scheme of the potential biological implementation of feedback learning.

In the following we give a more detailed description about changes in our manuscript in response to the reviewer's comments.

Major comments

Below are general comments that we ask authors to carefully consider and implement in the revised version of the manuscript.

1) As mentioned above, it is not clear why learning should necessarily depend on recurrent

changes within the decoded populations of neurons (or in this case, the RNN connectivity). Indeed, particularly for the case of WMP, changes in inputs may very well be able to drive the network state to the desired part of the pre-existing neural manifold to accommodate the BCI perturbation. In fact, following the original BCI study [Sadtler et al, 2014], the same group later reported [Golub et al, 2018] that animals adapt to WMP through a 'neural reassociation' strategy whereby pre-existing activity patterns are recycled and reassigned to new intended cursor movements. The authors of the present study do not currently discuss whether the mechanism they propose for WMP (through weight changes without any change in inputs) could lead to the same observations.
To address this important comment we implemented the analysis of Golub et al. 2018 to choose the hypothesis which best matches our simulated data (Fig.S13). We tested the three major hypothesis proposed in Golub et al. 2018: realignment, reassociation and rescaling. Interestingly, we found that our data best matches reassociation, similar to what has been observed experimentally, though we see minor differences due to the different nature of signals in our simulation and the experiment (much more variance due to closed-loop BCI control and more variable neural signals). We mention our results in a new paragraph in the Discussion section focussing on further experimental validation.

2) Another important point missing from the discussion is the fact that in the most recent BCI study cited [Oby et al, 2019], it was found that OMP learning was not simply the result of outside-manifold changes. A large fraction of the changes was in fact within-manifold (but in such a way that projections on the not-completely-orthogonal readout dimension could be adjusted). This result seems to be loosely consistent with the findings of the current study, and the authors should discuss whether their model predicts (or could accommodate) this type of solution for OMP learning.
Also here, we performed a new analysis mimicking the one used in Oby et al. 2019 to compare our simulation results with the experimental observations in Oby et al. 2019. We included a new supplementary figure (Fig.S14) showing that in our implementation of outside-manifold learning there are within-manifold contributions, in line with the experimental result. This finding is mentioned in the new paragraph in the Discussion section focussing on further experimental validation.

3) In the first part of the manuscript, where weight changes under ideal feedback are compared for WMP versus OMP, the authors make the point that weight change distributions are similar between the two perturbations. However, later in the manuscript, it is also stated that the dimensionality of these weight changes differs quite a bit between WMP and OMP (WMP leading to lower dimensional weight changes that OMP; Figure S4). Could that feature by itself already explain the difference in learning timescales between the two perturbations? Presumably, if weight changes are more correlated in WMP, they may occur more rapidly/easily, and may therefore already put WMP and OMP on an unequal footing even in the case of ideal feedback.
That's a very good point. We added more information about the dimensionality of weight change when we describe the weight change observed under ideal feedback learning (see screenshot below). Furthermore, we added a substantial part in the first paragraph of the Discussion section where we now also discuss that lower-dimensional weight changes can potentially also contribute to the experimental finding of Sadtler et al. 2014.

obtained by comparing the weights before and after the initial training phase. In addition to the amount of weight change, we also compared the effective rank and dimensionality of weight change between within- and outside-manifold relearning (Fig.S4). We found that weight changes happening during outside-manifold learning are slightly higher dimensional compared to weight changes during within-manifold learning. These results show that retraining within or outside of the original manifold does not necessarily lead to a different amount of weight change, yet weight changes related to within-manifold learning are slightly less dimensional.
[illegible]

4) Currently, there is no consideration of learning timescale, only learning feasibility, between WMP and OMP. That is, the model is judged based on its capacity to produce the desired output post retraining. Because the original BCI findings pointed to differences in learning feasibility as well as timescales between WMP and OMP, it seems relevant to assess the model in terms of timescale too. Concretely, do the number of trials needed to reach max performance for WMP systematically differ from OMP? This will most likely depend on the learning rule used (as seen in Figure S6), but it would be worth providing some data on this.
We added a supplementary figure providing information about the timecourse of the error and weight changes during relearning (Fig.S16). Under the considered learning algorithm, i.e. recursive-least-squares, there is no difference between within- and outside-manifold learning. We mention this in the Results section discussing relearning performance under perfect feedback (see screenshot below) and also again in the extended first paragraph of the Discussion section.

to a specific network initialization (Fig.2B), task setup (Fig.S1 & S5 & S19 & S20), or learning algorithm (Fig.S3 & S6). Comparing learning timescales did not show any difference for within- versus outside-manifold learning (Fig.S16), yet a more biologically plausible learning algorithm showed slightly slower learning for outside-manifold perturbations (Fig.S6). Together, these
[illegible]

5) One feature the authors do not currently rely on to constrain and further test their model is whether WMP or OMP learning would lead to an after-effect once the perturbation is removed. In the original BCI study, an after-effect was only seen after WMP, but not after OMP, presumably because no learning had occurred for this perturbation. Again, this seems consistent with what the authors of the present study claim (the fact that inferring feedback weights is only possible for WMP) and should be briefly mentioned in the discussion section.
We have now included a discussion about the match between our model and experimental observations concerning after-effects in the new paragraph in the Discussion section. Indeed, our results for within-manifold learning are in line with the experimental observation that the monkey has to relearn after switching back from a within-manifold perturbation. In contrast, our model currently does not provide a reason for why the monkey does not have to do this in the case of an outside-manifold perturbation. We showed that the inferred feedback weights could not be used for successful outside-manifold relearning, as they provide an erroneous error signal. Yet, in the current state of our model there is no fail-safe component, preventing learning under an invalid feedback signal.

6) It is stated that the initial performance decrements following WMP or OMP are similar (Figure 2B). Similarly, in the method section related to Figure 4, it is stated that the accuracy of the regression between network rates and x and y cursor velocities were "about the same" for WMP and OMP. How can this be? Since following an OMP, neural activity should have a smaller projection onto the new readout, shouldn't we expect an OMP to lead to minimal cursor movement after perturbation? In the method section, it is said that OMP perturbations are selected to achieve the same performance decrements as in WMP; doesn't that affect what types of OMP are indeed applied? I'm concerned that this biased procedure may generate OMP that no longer fit the features of OMP experienced by monkeys in the original BCI studies...

We thank the reviewer for pointing out that our description of perturbation selection was unclear. To address this we rewrote the description of how we selected within- and outside-manifold perturbations (see screenshot below). The produced OMPs have indeed small cursor velocities compared to WMPs, yet in terms of error they are matched.

In order to assure that both types of perturbations cause approximately the same performance error we ~~calculate the performance~~calculated the performance, measured as mean squared error, for 200 random perturbations of each type~~and then pick the ones which closely match in~~. Then we calculated the mean performance over all perturbations, rank them according to their difference to the mean and selected the perturbations closest to the mean. This ensured that the selected perturbations had similar mean squared error. Note that although the performance error ~~they cause.~~ was matched through this procedure, the cursor velocities for outside-manifold perturbations were scaled down compared to within-manifold perturbations. This is a consequence of the readout transformation to non-dominant directions in neural state space which naturally contain less variance and therefore lead to lower cursor velocities.

7) How does the regression procedure relating network dynamics to cursor movements work exactly? If the feedback learning is based on regressing activity against cursor movements, what this procedure gives is a set of target activities for every neuron. The authors should explain with an appropriate level of detail how these target activities are then turned into weight changes. It is not immediately obvious how the recursive-least-squares algorithm used in the ideal-observer case should be extended to work in the regression-based learning case. Moreover, it is unclear whether this procedure puts WMP at an advantage over OMP during retraining. If the regression is based on activity patterns that lie within a pre-existing manifold, wouldn't the regression be able to extrapolate and predict new cursor movements better within the manifold than outside?

We thank the reviewer for this very important comment, this was indeed unclear. By including another supplementary figure (Fig.S17) and rewriting text, we tried to clarify visually and verbally how the learned feedback weights through linear regression are used for recurrent relearning (Fig.S17C). The feedback weights are learned based on observing the network activity and cursor dynamics. For the relearning, the inferred weights are used not to propagate the observed or target cursor dynamics, but instead to propagate the error (= observed - target cursor dynamics), which is described in the Methods section. How recurrent weights are then adapted in response to an error on a neuron's activity (the propagated error signal) is given by the recursive-least-squares algorithm described in detail in the Methods section.

8) As I understand it, the rationale for adding noise on the ideal feedback signal, or making it partially available to the network, is to move away from biological implausibility. However, it is unclear how these conditions are in fact more realistic than the ideal case itself. The authors should better motivate the choices of 'lesions' they apply to the ideal feedback. Without those motivations, the various feedback conditions considered seem arbitrary.
To provide better motivation for our chosen imperfect feedback scenarios, we added a statement pointing out the noisy nature of neural signals in general, and the potential constraints in terms of number of long-range projections that could signal an error feedback.

9) As noted by the authors, a more realistic version of the model is one where weight changes need to be inferred, as opposed to being fed to the network. How viable is the process of inferring weights from regressing the internal dynamics against cursor velocities in an actual network of neurons? It is not immediately clear what makes this strategy any more viable than the ideal feedback strategy. The fact that inferred weights deviate from the ideal weight changes is not sufficient to conclude that the former are more realistic.
We agree with the reviewer that learning a feedback signal via linear regression is not directly a more biologically plausible scenario. To address this, we showed in an extra supplementary figure (Fig.S17) that linear regression can be implemented by a simple biological learning rule. Using this alternative implementation of feedback learning does not change the accuracy of the inferred feedback weights (Fig.S17B). This is also mentioned in a new paragraph at the end of the feedback learning section discussing potential biological implementation of our setup.

Minor comments

1) Throughout the manuscript, one result that seems to come up at multiple occasions is the fact that a perfect manifold alignment is not required for performance in the task to max out. It is stated indeed that ~40% of overlap is generally enough between the readout and the intrinsic manifold to obtain high performance. To what do authors attribute this finding? Do they think it is also the case in a real BCI setting? How general is this number of 40%?
We added an additional panel in Fig.2D where we show that the required overlap for outside-manifold learning is dependent on the BCI manifold dimensionality. Furthermore, we added the following sentence to the result section describing this figure (see screenshot below).

task. Instead it is enough to have a sufficient amount of overlap, or match the most prominent modes. The amount of necessary overlap likely depends on a combination of task dimensionality [Gao et al., 2017] and BCI manifold dimensionality (Fig.2D inlet).

Also in the real BCI setting it has been found that there are major contributions lying within the original manifold, yet contributing to outside-manifold learning (Oby et al.). Therefore, also in this case the overlap with the newly imposed BCI manifold would be less than 100%.

2) Currently the caption of Figure 4G does not explain what the black line corresponds to (it is only explained in the method section). Please add it in the caption for clarity.
We added a sentence in the caption of Fig.4 (see screenshot below).

$1 + (\sum \lambda_i)^2 / \sum \lambda_i^2$ where λ_i are the Eigenvalues of the covariance matrix. The data points show feedback learning results for 20 simulations, whereas the black lines are interpolations between OMP and initial BCI mappings for which we evaluated feedback learning.

3) Regarding the effect of noisy feedback (Figure 3B): is the difference between WMP and OMP robust across features of the model? The fact that WMP does better compared to OMP only in an intermediate range of noise makes it questionable whether it is not just an oddity of the model under the current parameter values.

To test whether our results for learning under a noisy feedback signal are robust we reran simulations using the alternative, more biologically-plausible, learning rule proposed in in Fig.S6. The result that within-manifold learning is more robust for a given noise strength was holding (Fig.S18).

4) I suggest clarifying the section on the relationship between OMP learning and the dimensionality of neural dynamics. This part would benefit from stating explicitly the goal of the analysis, which is to analyze how OMP learning differs for varying degrees of dimensionality. Moreover, the overall logic of the argument developed in that section is hard to follow. If I understand correctly the argument, in a high-D system, the variance is more evenly distributed across dimensions, which means that dimensions are all roughly equivalent, and therefore do not suffer too much from shuffling. Whereas in low-D, because the space is highly anisotropic, shuffling does impact performance more dramatically. Finally, I am unsure what the following sentence means: “the inferred feedback weights for an outside-manifold perturbation in our main simulation setup always pointed in directions which are within the original manifold”.

We highly appreciate this comment and we agree that the explanation for the feedback learning results was inconclusive. In order to improve this we created a new supplementary figure (Fig.S12). It shows as new analysis supporting our explanation paragraph in the main manuscript (now rewritten too). The main goal of this paragraph was not to primarily analyse OMP for varying dimensionality of neural dynamics, but instead to understand why outside-manifold feedback learning fails in the considered case. Hopefully this point comes across more clearly now.

5) Would the results hold if the target is given to RNN as a tonic input (which more directly match the experimental setting) and not as a pulse?
To test this, we adapted our simulation to work with a tonic, instead of a pulsed, input signal. The simulation results are shown in an additional supplementary figure (Fig.S19), where we show that we obtain nearly identical results compared to our simulations in the main paper.

6) An important point noted in the paper is “… our study gives a new perspective on the general problem of credit assignment, proposing that correct feedback learning critically depends on the alignment between output transformation and internal manifold in a specific neural pathway.” I missed how this was shown in the paper.

We refer here to the results in Figure 4G, which shows the dependence of feedback learning accuracy on alignment between BCI readout and internal manifold. The reference

to the relevant figure was added in the manuscript.

7) The solution in the weight space for either type of learning is not unique. For example, the solution found with pseudoinverse may be different from regression feedback and other learning strategies. Therefore, it is unclear how weigh-change measure in Figure 2 is relevant to a more biological learning. This is quite evident in Figure 2 where there are several instances of high performance but low manifold overlap indicating that the solutions under different retraining schemes and perturbations may not converge to the same solution, and are thus not necessarily comparable. The results shown simply indicate that there exists a solution with similar distance but doesn't guarantee that those solutions are as easily reachable through all learning mechanisms. Maybe, in the space of reachable weight states the distance is a lot longer for off-manifold. This needs some thinking and discussion.
We appreciate this interesting comment and tried to address it in the first paragraph of the Discussion section, where we discuss the 'meaning' of the weight change analysis. We agree that our results about weight change can not predict directly how much weight change a biological system would have to undertake. We argue instead, that due to the nature of the used learning algorithm, the observed weight change can potentially serve as an upper bound. We also observed that a more biologically-plausible learning rule (Fig.S6) leads to much smaller weight changes in general, yet produces a similar relearning performance.

8) The text is really opaque with respect to P-OMP. In fact, there is no mention of this despite it reappearing several times in figures.

We were not totally sure that we understood this comment correctly. The overlap between manifold after outside-manifold learning and the perturbed BCI manifold is discussed at various points in the last Results section on incremental outside-manifold learning. P-OMR serves as abbreviated label for this. We are very happy to address this concern if the reviewer can give us more details.

Reviewer #2: Animal experiments show that in a BCI task outside-manifold remapping (OMR) are much difficult (if not impossible) to learn than within-manifold remapping (WMR) (Sadtler et al. 2014). In this manuscript authors address the issue of why there maybe a difference in the OMR and WMR. This question has been previously addressed by Waernberg and Kumar (PloS Comp 2019), where they showed that OMR may require a larger change in local connectivity than WMR. Similarly Menendez and Latham (2019) argued that the learning difficulty can be attributed to inputs. In this study authors provide a different explanation.
They have uses a recurrent rate network (RNN) that learns with an online recursive-least-square algorithm. In its initial formulation this algorithm requires the pre-existence of carefully tuned feedback weights, (not biologically plausible). The authors address this by showing that a) these feed-back can be noisy or sparse (Fig 3), b) the feed-back weights can be learned (Fig 4) and c) that a similar result can be found also with a local learning rule (Fig S6). They argue that only when feedback error signal is noisy that OMR is difficult. They show that when ideal-observer feedback is available there is no fundamental difference between WMR and OMR. When such ideal feedback is not available (as would

be the case in a biologically plausible scenario), WMR outperforms OMR -- i.e. WMR is more robust to noise in the error signal.

Whether "it is possible to infer useful feedback weights by regressing neural activity against cursor dynamics" in the brain, is another discussion -- but I think this paper points to a variable that may control learning in neural networks -- at least those using supervised learning.

While the manuscript is very well written and illustrated with excellent graphics, there are few points that need to be addressed in a revised version.

- In contrast to the conclusion of Waernberg+Kumar paper, the present study shows that there is no difference in amount of local network rewiring required for OMR and WMR. After having read the paper I still do not understand why this should be the case? Unfortunately the current manuscript does not provide any intuitive or conceptual understanding of this. I wonder if authors can provide a more intuitive way to think about this. Authors try to address this question on page 8. But I don't understand why 'wrong Eigenvectors' should be qualitatively different for low and high dimensional dynamics? I think point needs some more explanation.
This is an important point and it was not clear in the paper previously. To address this, we added an additional supplementary figure describing in detail the difference with the Waernberg+Kumar paper (Fig.S15). We reimplemented their network and found out that the exact difference is due to the specific way Waernberg+Kumar implemented the BCI perturbation. We rewrote the paragraph in the Discussion section and tried to clarify the difference in regard to these new findings (see screenshot below).

A recent computational study has shown that weight changes necessary for within-manifold learning are much smaller compared to outside-manifold learning and proposed this finding as a potential reason for the experimental observation in Sadtler et al. [Wärnberg and Kumar, 2019]. In contrast, we could not observe a difference in weight change during retraining (Fig.2C). However, we did observe that, if we constrain the number of plastic weights, within-manifold learning is more successful compared to outside-manifold learning (Fig.3C-D) and also that weight changes related to outside-manifold learning are slightly higher-dimensional (Fig.S4C& D). One fundamental difference compared to ~~Wärnberg~~ Waernberg and Kumar is that ~~, in our study, the weight dynamics are not constrained to one dimension. Instead , by calculating the effective rank of the weight change during relearning we found that weight dynamics explore various dimensions (see Fig.S4). Having a more flexible training algorithm, which is potentially able to explore all weight configurations, could explain why we found that small weight adaptations are sufficient for successful~~ they used an alternative way of implementing the BCI perturbation, which led to the finding that the weight changes are bigger during outside-manifold learning (Fig.S15). Instead of setting new target readout dynamics, which could be derived given a certain within- ~~as well as~~ or outside-manifold ~~learning~~perturbation, they implemented a perturbation by changing the encoder matrix (called K in their case). As the encoder matrix injects the readout dynamics back into the system, this already changes the effective recurrent weights in the network, even before retraining. To counteract the perturbation, the readout weight matrix Φ is retrained to produce the same readout dynamics as initially. The overall weight change which Waernberg and Kumar reported in their paper is given by an outer product of both matrices $K\Phi$. By reimplementing their simulations, we replicated their finding that within-manifold learning leads to smaller weight changes than outside-manifold learning (Fig.~~Which one of these two scenarios is more plausible in a biological context is unclear. Thus, any conclusions drawn critically depend on further investigation of biological learning~~

~~dynamics~~S15C&E&G). Interestingly though, when we implemented the BCI perturbation by setting new target readout dynamics, instead of changing K itself, the observed weight changes were small both for within- and for outside-manifold learning (Fig.S15D&F&H). Thereby, we could identify the main difference between both studies and replicate our finding that within- and outside-manifold learning produces similar amount of weight change if retraining starts from the initial connectivity, as it would be for a BCI monkey experiment.

As having a feedback signal is a general concept of motor control [Kawato, 1999, Wolpert

- In Fig S4 (which I think should be in the main text) author show the weights before and after BCI perturbations. Here authors show the effect rank of the dW matrix in different conditions. But I don't think it makes sense to compare the effect rank of dW with sparse rank-1 matrix. I think the comparison should be made with a sparse rank-2 because the task (and therefore dW) has 2 dimensions. My guess is that the effect rank of such a matrix would be comparable to that if dW_OMR and dW_WMR.

That’s an interesting point and we indeed saw that adding a sparse rank-2 matrix as control is closest to the effective rank measured for within- and outside-manifold learning. We added this in Fig. S4, such that it now shows the two ‘extremes’ (rank-1 and full rank),

but also the more relevant comparison to a sparse rank-2 matrix. We added two sentences about the dimensionality of weight changes in the main paper (see screenshot below).

[illegible]
obtained by comparing the weights before and after the initial training phase. In addition to the amount of weight change, we also compared the effective rank and dimensionality of weight change between within- and outside-manifold relearning (Fig.S4). We found that weight changes happening during outside-manifold learning are slightly higher dimensional compared to weight changes during within-manifold learning. These results show that retraining within or outside of the original manifold does not necessarily lead to a different amount of weight change, yet weight changes related to within-manifold learning are slightly less dimensional.
In order to understand how our network achieves high performance after the adaptation phase,

- It would be fair to mention the Waernberg and Kumar 2019 and Menendez and Latham (2019) already in the introduction. After all they have addressed the question by varying the local connectivity (Waernberg and Kumar) and by changing the inputs (Menedndez and Latham).
That's very true. We included another paragraph in the Introduction describing the previous work of Waernberg+Kumar and Menendez+Latham (see screenshot below).

Previously, two computational studies aimed at uncovering the mechanisms underlying the constraints on learning in the described experiment. Waernberg & Kumar used a simplified implementation of the BCI task to compare the amount of weight change which is required for within- and outside-manifold learning. They found that within-manifold learning requires less weight change in their simulations and thereby concluded that within-manifold learning is faster because of smaller underlying synaptic changes [Wärnberg and Kumar, 2019]. In contrast, Menendez & Latham investigated the possibility that learning results from a change in input to motor cortex [Menendez and Latham, 2019]. Although there have been these two attempts to explain the experimental observations in Sadtler et al., the search of underlying neural mechanisms remains inconclusive.

- Authors wrote that "one fundamental difference compared to Waernberg and Kumar is that, in our study, the weight dynamics are not constrained to one dimension (p. 13)." This statement is not quite correct, In Waernberg+Kumar paper the weight "dynamics" was constrained to D dimensions where D is the dimensionality (> 1) of the intrinsic manifold and in sparse matrices, the matrix rank was much higher than the dynamics.

The whole paragraph describing the difference with the Waernberg+Kumar paper was rewritten, as the difference became clearer after reimplementing their simulations (Fig.S15). Hopefully it now provides a clear and concise explanation for the observed differences between their and our study.

- Predictions: The first prediction says "for within-manifold perturbations, it is possible to infer useful feedback weights by regressing neural activity against cursor dynamics" This is not really a prediction -- this is what authors have shown to be the case in their model. Evidently, the model has many non-biological features including the learning rule, so just because something is seen in an RNN does not mean it is a prediction for the brain. Also, the second prediction should be reformulated in view of the paper by Oby et al. (2020).
We agree that it is not necessarily the case that results we obtain by analyzing an artificial

learning system can be transferred to the real brain. Nevertheless, we think that by testing if the ‘artificial’ feedback learning via linear regression could be done using real, in contrast to simulated data, we could gain further evidence on whether our proposed implementation of feedback learning (Fig.S17) has any chance to perform well in reality. Therefore, we think that the prediction that correct feedback weights (corresponding to the pseudo-inverse of the BCI mapping) can be inferred by using real neural data and cursor dynamics is a prediction which we hope will be tested experimentally. Concerning the second part of the comment, we unfortunately did not understand how we should relate the results of Oby et al. 2020 to our second prediction “...learning performance during a BCI task can be predicted by the alignment between BCI readout and internal manifold of the connected brain region…”. We are very happy to address this concern if the reviewer can give us more details.

- How was the MSE scaled? In figure S2A and S2E it looks like a typical error would be on the order of 0.2 for untrained networks. In Fig 2B and similar figures the error is around 1.0. In Fig S3 the scale is different for panels C and D, and different from the scale in the main figures?

We thank the reviewer for noticing this discrepancy in the scaling of the MSE. We addressed this comment by adding a sentence describing the exact calculation of MSE in the Methods section. To measure training performance, the mean squared error is calculated in each of 50 test trials and then summed across test trials. In all plots where we show learning curves, the MSE is not summed over 50 test trials, but instead over all update steps in one given training trial (which is 90). We added a note on this difference in the caption of Fig.S6 and Fig.S16. In Fig S3, we describe a slightly different simulation used for the Pytorch implementation. The target values are higher than in the main paper and the MSE in Fig.S3C and Fig.S3D is not summed over trials or time steps here, which we also denoted now in the caption.

- The sentence ”In order to assure that both types of perturbations cause approximately the same performance error we calculate the performance for 200 random perturbations of each type and then pick the ones which closely match in the performance error they cause.” is a bit unclear. How was this matching done? And does this mean the MSEs for WMP and OMP were equal by design in for example Fig 2B? Were these errors similar on average even without this matching?
To address this comment we added a extended description of how we select within- and outside-manifold perturbations (see screenshot below).

In order to assure that both types of perturbations cause approximately the same performance error we ~~calculate the performance~~calculated the performance, measured as mean squared error, for 200 random perturbations of each type~~and then pick the ones which closely match in~~. Then we calculated the mean performance over all perturbations, rank them according to their difference to the mean and selected the perturbations closest to the mean. This ensured that the selected perturbations had similar mean squared error. Note that although the performance error ~~they cause.~~ was matched through this procedure, the cursor velocities for outside-manifold perturbations were scaled down compared to within-manifold perturbations. This is a consequence of the readout transformation to non-dominant directions in neural state space which naturally contain less variance and therefore lead to lower cursor velocities.

- In the current formulation, the network equations are deterministic, i.e. has no noise. How important is this?

This is an interesting point. To address it we reran simulations where we added normally distributed, centered, noise to each neuron independently (additional supplementary figure Fig.S20). We varied the amount of noise by varying the standard deviation (which is f in the figure). We found that our results for within- and outside-manifold learning are holding when noise is added in the system. Of course, beyond a certain noise level it is no longer possible to train the network at all (Fig.S20E-F).

Minor:

- It is unclear whether the performance error e^P is a scalar or a vector. In the text, it says that is a mean squared error, suggesting that it is a scalar. But in the equation e = W^{fb}e^P it seems it should be a 2D vector, because W^{fb} is the pseudo-inverse of the 2xN read-out matrix T.
- I think a minus sign missing in the equation for dP? Laje & Buonomano (2013), Nicola & Clopath (2017) and Waernberg & Kumar (2019) all have a minus sign in the corresponding equation.
- There is no index j on the right-hand side of the equation for dW. I'm guessing one of the ls in the nominator should be a j?
- The notation dW and dP might suggest a continuous rate of change (like d/dt) rather than a step-wise update. Perhaps use Δ instead of d if it's a fixed update happening every (second) time step?
- The variable T is used for both trial duration and BCI transformation.

All of the results concerning the Methods section were included in the new manuscript (see screenshot below).

To implement learning in the network we ~~use~~ used an adapted form of the recursive-least-squares algorithm, which ~~is~~ was inspired from [Laje and Buonomano, 2013]. If learning is on, it ~~starts~~ started after the target cue period. Then, at every second time point (which corresponds to ~~every $20ms$~~ $\Delta t = 20ms$) an update step ~~is~~ was made. Firstly, the performance error e^P, given by the ~~mean squared error between the target~~ difference between the produced cursor velocity and the ~~produced~~ target cursor velocity, ~~is~~ was calculated. Via a linear transformation this ~~performance error is~~ two-dimensional performance error was then assigned to errors e on the activity of single units in the network.

$$e = W^{fb} e^P$$

This transformation W^{fb} is what we ~~define~~ defined as feedback weights. In the ideal-observer case, this feedback matrix ~~is~~ was given by the pseudo-inverse of the BCI transformation. Note that we ~~calculate~~ calculated the error for each neuron through these feedback weights, which ~~is~~ was used to adapt recurrent weights in the network, but there ~~is~~ was no additional input for each neuron due to this error signal. The recurrent weight update is given by

$$dW\, W_{ij}(t) = W_{ij}(t-\Delta t) - \frac{e_i \sum_k P_{ik} r_k}{1 + \sum_l r_l \sum_k P_{lk} r_k} \frac{e_i(t) \sum_k P^i_{jk}(t) r_k(t)}{1 + \sum_m \sum_n r_m(t) P^i_{mn}(t-\Delta t) r_n(t)}$$

where e_i is the error for unit i and ~~P~~ P^i is a matrix which estimates the inverse of the correlation matrix ~~. P is~~ of the input to neuron i. P^i was also updated in every update step and the update rule is ~~, in matrix form, given by~~ given by

$$dP\, P^i_{jk}(t) = \frac{(Pr)(Pr)^T}{1 + r^T P r} P^i_{jk}(t-\Delta t) - \frac{\sum_m \sum_n P^i_{jm}(t-\Delta t) r_m(t) r_n(t) P^i_{nk}(t-\Delta t)}{1 + \sum_m \sum_n r_m(t) P^i_{mn}(t-\Delta t) r_n(t)}$$

At the start of learning, the ~~matrix P is~~ matrices P^i were initialized as diagonal ~~matrix~~ with 0.05 on the diagonal. For each training period the network ~~is~~ was trained for 80 trials. Note that the training algorithm ~~does~~ did not change the structural connectivity, so only connections which ~~are~~ were non-zero at the beginning ~~are~~ were updated.

- The forward slash in the x-label of Fig 2C might be read as division at first glance.

We changed the x-label in Fig.2C and removed the division sign.
